# Cdk5 Is Required for Memory Function and Hippocampal Plasticity via the cAMP Signaling Pathway

**DOI:** 10.1371/journal.pone.0025735

**Published:** 2011-09-30

**Authors:** Ji-Song Guan, Susan C. Su, Jun Gao, Nadine Joseph, Zhigang Xie, Ying Zhou, Omer Durak, Lei Zhang, J. Julius Zhu, Karl R. Clauser, Steven A. Carr, Li-Huei Tsai

**Affiliations:** 1 Picower Institute for Learning and Memory, Massachusetts Institute of Technology, Cambridge, Massachusetts, United States of America; 2 Department of Brain and Cognitive Sciences, Massachusetts Institute of Technology, Cambridge, Massachusetts, United States of America; 3 Howard Hughes Medical Institute, Massachusetts Institute of Technology, Cambridge, Massachusetts, United States of America; 4 Stanley Center for Psychiatric Research, Broad Institute, Cambridge, Massachusetts, United States of America; 5 Department of Pharmacology, University of Virginia, Charlottesville, Virginia, United States of America; Université Pierre et Marie Curie-Paris6, INSERM, CNRS, France

## Abstract

Memory formation is modulated by pre- and post-synaptic signaling events in neurons. The neuronal protein kinase Cyclin-Dependent Kinase 5 (Cdk5) phosphorylates a variety of synaptic substrates and is implicated in memory formation. It has also been shown to play a role in homeostatic regulation of synaptic plasticity in cultured neurons. Surprisingly, we found that Cdk5 loss of function in hippocampal circuits results in severe impairments in memory formation and retrieval. Moreover, Cdk5 loss of function in the hippocampus disrupts cAMP signaling due to an aberrant increase in phosphodiesterase (PDE) proteins. Dysregulation of cAMP is associated with defective CREB phosphorylation and disrupted composition of synaptic proteins in Cdk5-deficient mice. Rolipram, a PDE4 inhibitor that prevents cAMP depletion, restores synaptic plasticity and memory formation in Cdk5-deficient mice. Collectively, our results demonstrate a critical role for Cdk5 in the regulation of cAMP-mediated hippocampal functions essential for synaptic plasticity and memory formation.

## Introduction

The hippocampus is considered to be a key region for long-term memory formation in humans and rodents [1], [2], yet the molecular mechanisms underlying memory formation are still not fully understood. Transgenic mouse studies using hippocampal region-specific knockout of the NMDA receptor NR1 subunit strongly support the hypothesis that synaptic plasticity, especially NMDAR-mediated synaptic plasticity, is crucial for normal learning and memory [3], [4]. Numerous genetic and molecular studies have revealed that NMDAR activation, and its downstream cascade of events, are critical for synaptic plasticity. These events include calcium entry, autophosphorylation of CaMKII, activation of protein phosphatases, and the relocation and modification of AMPA receptors [5], [6], [7]. Perturbations in the molecular cascade downstream of the NMDAR pathway result in defects in both long-term potentiation (LTP) and memory formation. Blocking the NMDAR pathway, in addition, impacts long-term depression (LTD). Interestingly, the PKC gamma mutant mouse, which displays normal LTD and impaired LTP, exhibits a relatively mild behavioral deficit [8]. Thus, both forms of synaptic plasticity (LTD and LTP) are required for memory formation.

The cyclic AMP (cAMP) pathway is also critically involved in synaptic plasticity and learning and memory. The second messenger cAMP, as well as the cAMP-dependent protein kinase A (PKA), have been implicated in short- and long-lasting synaptic plasticity and intrinsic neuronal excitability in *Aplysia* by activating cAMP-responsive element binding protein (CREB)-dependent transcription [9]. Accumulating data regarding the molecular events underlying CREB-dependent learning and memory in *Drosophila*, mice, and rats all indicate that CREB activation by phosphorylation at the Serine 133 residue is required for the maintenance of LTP and formation of long-term memory [10]. The maintenance of LTP and long-term memory are also both dependent on PKA activity and CREB-mediated transcription [11]. Cyclic nucleotide phophodiesterases (PDEs) catalyze the conversion of cAMP and cGMP into AMP and GMP, respectively, and are important for the homeostatic regulation of cyclic nucleotide-mediated signaling events. The PDEs are grouped into several families, two of which, PDEs 4 and 7, exclusively target cAMP. Rolipram, a PDE4-specific small molecule inhibitor, has antidepressant effects [12] and was recently found to facilitate memory formation and synaptic plasticity in rodents [13]. Rolipram was also shown to reverse synaptic plasticity and memory deficits in CBP+/− mice, a mouse model of Rubinstein-Taybi syndrome [14], [15].

Cyclin-dependent kinase 5 (Cdk5) was initially classified as a cyclin-dependent kinase based on sequence homology to other Cdks that operate in the cell cycle [16]. Cdk5 has been implicated in almost every aspect of brain development and neural function including neuronal migration, neurite extension, synaptic transmission, and synaptic plasticity [17], [18], [19], [20], [21]. Intriguingly, Cdk5 gain-of-function mutations result in an increased number of synapses *in vivo* and the facilitation of LTP in slices [20], whereas the loss of Cdk5 activity in p35 knockout mice results in impaired hippocampal LTD [22]. In contrast, an inducible Cdk5 conditional knockout mouse model exhibited facilitated LTP and enhanced memory formation via reduced degradation of the NR2B subunit of the NMDA receptor [23]. Despite a greater understanding of how Cdk5 is critically important for synapse formation, the *in vivo* role for Cdk5 as a part of a signaling pathway crucial to hippocampal learning and memory remains unclear.

In the current report, we found that Cdk5 loss of function in distinct hippocampal circuits resulted in impairments in various memory functions and synaptic plasticity, observations distinct from previously reported findings using a different Cdk5 mouse model [23]. In addition, multiple PDE isoforms were markedly upregulated, which led to the dysregulation of cAMP pathway and impaired CREB phosphorylation. Remarkably, the observed deficiencies in Cdk5 conditional knockout mice can be rescued by rolipram treatment. Taken together, these results indicate a key function for Cdk5 in regulating cAMP signaling via modulation of PDE expression to facilitate synaptic plasticity and hippocampal-dependent memory formation.

## Results

### Associative and spatial memories are impaired in Cdk5f/f/T29 mice

**Figure 1:**
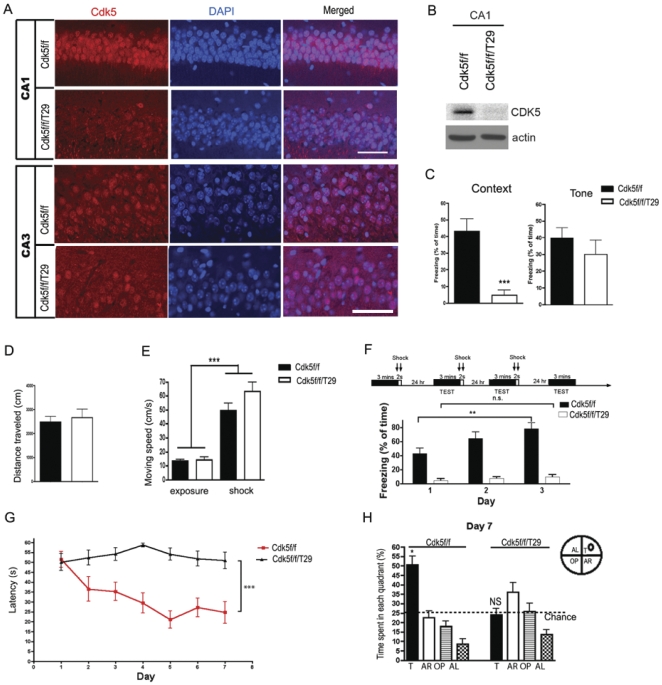
Associative and spatial learning are impaired in Cdk5f/f/T29 mice. **A**. Immunostaining for Cdk5 in hippocampal areas CA1 and CA3 in Cdk5f/f, Cdk5f/f/T29 mice. Scale bars, 100 µm. **B**. Western blot showing the reduction of Cdk5 in micro-dissected area CA1 of Cdk5f/f/T29 mice. **C**. Context- and tone-dependent (cued) fear conditioning tests for Cdk5f/f/T29 and Cdk5f/f (control) mice. Cdk5f/f, n = 16; Cdk5f/f/T29, n = 16 for context; Cdk5f/f, n = 8; Cdk5f/f/T29 n = 8 for tone-dependent task. **D**. Distance traveled during the initial 3 min exposure to the training box. **E**. The velocity during the 3 min of training and electrical foot shock. **F**. In context-dependent fear conditioning test, Cdk5f/f and Cdk5f/f/T29 mice were trained for 3 continuous days. Cdk5f/f, n = 8; Cdk5f/f/T29, n = 8. **G**. Morris water maze test. Escape latencies of Cdk5f/f mice improved significantly faster than Cdk5f/f/T29 mice. N = 8 for each group. **H**. On day 7, a probe trial was conducted in which the swimming time in each quadrant was quantified. T, target quadrant; L, left quadrant; O, opposite quadrant; R, right quadrant. *p<0.05; **p<0.005; ***p<0.001.

To evaluate the consequences of Cdk5 ablation in hippocampal neurons, Cdk5f/f/T29 mice were generated using the Cre line T29-2 [24], [25], in which Cre is highly expressed in CA1 pyramidal neurons of the hippocampus (**Table 1** **,** **Figures 1A, B** **, S1A–D**), and subjected to various behavior tasks. The ablation of Cdk5 using T29-Cre does not lead to significant changes in overall brain architecture or cell survival (**Figure S1F**). We noted in the T29-Cre line that Cre-mediated Cdk5 knockout is relatively specific to area CA1 in young mice (2.5–3.5 months old), with the deletion of Cdk5 spreading to cortical and other brain regions in older (4 month-old) mice. Beginning at 5 months of age, Cdk5f/f/T29 mice suffered mild to severe seizures and died by 8 months, which might be due to the spreading of T29-Cre expression [26], [27]. Therefore, we used 2.5 to 3.5 month-old mice to perform all behavior tests. Cdk5f/f/T29 mice were trained using Pavlovian fear conditioning paradigm 24 hours prior to a memory test. Cdk5f/f/T29 mice exhibited significantly reduced freezing behavior compared to control littermates (Cdk5f/f) in the context-dependent memory test (**Figure 1C**). Consistent with the notion that the hippocampus is not required for cued fear conditioning, Cdk5f/f/T29 mice did not exhibit significantly different freezing behavior compared to controls in the tone-dependent memory test (**Figure 1C**). Moreover, the reduced freezing behavior in Cdk5f/f/T29 mice was not due to motor defects or impaired pain sensation, as their locomotor activity and response to electrical foot shock were similar to control mice (**Figure 1D–E**). No significant differences in anxiety levels were detected using the open field test (**Figure S1G**). Notably, after repeated fear conditioning training, control Cdk5f/f mice exhibited increased freezing behavior after three days of training, while Cdk5f/f/T29 mice demonstrated no increase in freezing behavior (**Figure 1F**). These results suggest that memory consolidation is impaired in the Cdk5f/f/T29 mice.

**Table 1:** Cdk5 mutant mouse strains generated for analysis.

MOUSE STRAIN	CRE PROMOTER	CDK5 KNOCKOUT REGION
Cdk5f/f/T29	CaMKIIα (T29-1)	Restricted mainly to hippocampal area CA1
Cdk5f/f/KA1	Kainate receptor-1	Restricted mainly to hippocampal area CA3
Cdk5f/f/CW2	CaMKIIα (CW2)	Restricted to forebrain excitatory neurons

The mouse strains used for analysis include three genotypes: Cdk5f/f/T29, Cdk5f/f/KA1, and Cdk5 f/f/CW2. Three different Cre promoter lines were used to generate these genotypes by breeding the Cre line to the Cdk5 floxed lines to obtain the CA1, CA3, and forebrain-specific knockdown of Cdk5. In all experiments, 2.5–3.5 month old mice were used, and age-matched littermate Cdk5f/f mice were used as controls.

We next utilized the Morris water maze hidden-platform paradigm to evaluate hippocampal-dependent spatial learning in the Cdk5f/f/T29 mice. Over the course of the training period, Cdk5f/f mice displayed a significant decrease in the latency to find the hidden platform (51.5±4.1 s, day 1; 24.7±5.5 s, day 7). However, the Cdk5f/f/T29 mice consistently showed longer escape latencies during the training period that did not improve over time (50.1±4.3s, day 1; 51.0±4.2 s, day 7; control *vs.* KO group, *p*<0.0001, one way ANOVA; **Figure 1G**). During the probe trial on day 7, when the platform was removed from the swimming pool, Cdk5f/f/T29 mice spent a comparable duration in the four quadrants, while the Cdk5f/f mice spent significantly more time in the target quadrant (T), where the platform had previously been placed (**Figure 1H**). The decreased time spent by the Cdk5f/f/T29 mice in the target quadrant compared to Cdk5f/f was not due to motor defects, as the swimming speed of Cdk5f/f/T29 mice was similar to Cdk5f/f mice (**Figure S1H)**. Thus, the ablation of Cdk5 in CA1 pyramidal neurons significantly impairs spatial learning.

To confirm these results in another mouse model, we used the forebrain-targeted Cre line [28] (CW2 line; CaMKII promoter transgenic mice, **Figure S1C, E**) to generate forebrain-targeted Cdk5 deletion in excitatory neurons (**Figure S1I**). Consistent with the Cdk5f/f/T29 mice, the Cdk5f/f/CW2 mice displayed impaired spatial learning in the Morris water maze task and contextual fear conditioning (data not shown). Our data strongly suggest that ablation of Cdk5 in excitatory neurons impairs memory formation without apparent neuronal death (**Figure S2**).

### Pattern completion-based memory retrieval is impaired in Cdk5f/f/KA1 mice

**Figure 2:**
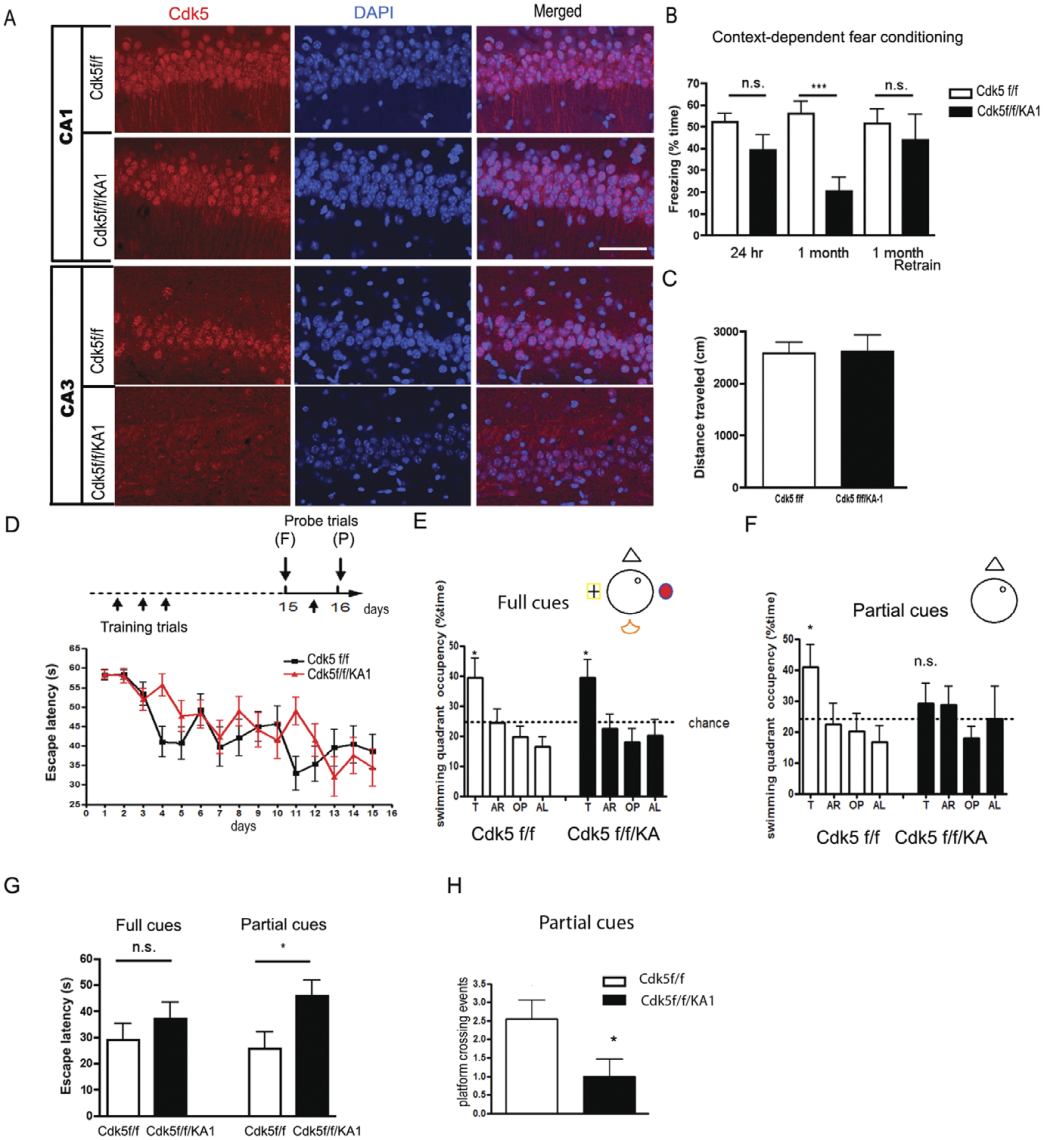
Memory retrieval is impaired in Cdk5f/f/KA1 mice. **A**. Immunostaining for Cdk5 in hippocampal areas CA1 and CA3 in Cdk5f/f and Cdk5f/f/KA1 mice. Scale bar, 100 µm. **B**. Contextual-dependent fear conditioning test for Cdk5f/f/KA1 mice. Cdk5f/f, n = 10; Cdk5f/f/KA1, n = 8. **C**. Distance traveled during the initial 3 min exposure to the training box. **D**. Morris water maze training paradigm (top). Mice were trained for 16 days. On day 15, they were tested in a probe trial with full visual cues around the tank, followed by an additional training trial. On day 16, mice were tested in another probe trial with most of the visual cues removed around the tank. (Bottom) Latencies for the mice to reach the platform during training did not show significant differences between Cdk5f/f/KA1 (n = 10) and Cdk5f/f mice (n = 10). (P), partial cues, (F), full cues. **E**. Cdk5f/f/KA1 mice showed similar preferences toward the target quadrant as the Cdk5f/f group in a full cue trial on day 15. **F**. Cdk5f/f/KA1 mice did not show preferences toward the target quadrant in the partial-cue-trial on day 16. **G**. Cdk5f/f/KA1 mice take more time to reach the hidden platform position than Cdk5f/f mice in the partial cue, but not the full cue, trial. **H**. In the probe trial with partial cues, the average number of crossings over the platform position is much lower for the Cdk5f/f/KA1 group than that for the Cdk5f/f group. *p<0.05; **p<0.005.

To evaluate Cdk5 function in other neuronal populations, we utilized a kainate receptor subunit KA1 promoter Cre line (G32-4) [29], which expresses Cre mainly in CA3 pyramidal neurons, to generate conditional Cdk5 KO mice (Cdk5f/f/KA1) (**Figure 2A** **, Figure S3A–C**). In contrast with Cdk5f/f/T29 mice, no seizure activity was observed in these Cdk5f/f/KA1 mice (data not shown). Therefore, 2.5–3.5 month-old Cdk5f/f/KA1 mice were subsequently used for behavior tests. The Cdk5f/f/KA1 mice did not show significant differences in freezing behavior 24 hr after contextual fear conditioning training compared to littermate (Cdk5f/f) controls (52.2±3.9% in Cdk5f/fs *vs.* 39.2±6.9% in Cdk5f/f/KA1; **Figure 2B**). However, upon being tested in the training chamber one month after the training, Cdk5f/f/KA1 mice exhibited dramatically reduced freezing behavior compared to the Cdk5f/f mice (55.9±5.8% in Cdk5f/fs *vs.* 20.5±6.1% in Cdk5f/f/KA1). This result suggests that either long-term memory consolidation or long-term memory retrieval is impaired in the Cdk5f/f/KA1 mice. This observation cannot be attributed to the loss of the ability to express freezing behavior, because further training of these mice in the contextual fear conditioning chamber led to equal freezing behavior of Cdk5f/f/KA1 and Cdk5f/f mice when tested 24 hr after an additional training (1 month re-train group, **Figure 2B**). The behavioral phenotype is not due to alterations in moving ability (**Figure 2C**).

The Cdk5f/f/KA1 mice were then tested with the Morris water maze hidden-platform task. During the fifteen days of training, the Cdk5f/f/KA1 mice exhibited similar spatial learning behavior to the Cdk5f/f littermates (**Figure 2D**). The average escape latencies did not differ between the two groups (Cdk5f/f/KA1, 58.3±1.1 s (day 1) to 34.5±4.8 s (day 15); Cdk5f/f 58.3±1.3 s (day 1) to 38.6±4.4 s (day 15)). The probe trial revealed that the Cdk5f/f/KA1 mice display similar searching patterns as the control littermates, spending more time in the target quadrant when presented with full spatial cues (**Figure 2E**). However, when presented with partial spatial cues during the probe trial (day 16), the Cdk5f/f/KA1 mice did not spend significantly more time in the target quadrant (**Figure 2F**). When presented with partial cues in the hidden platform test, the Cdk5f/f/KA1 mice displayed significantly increased escape latencies (45.7±5.9 s) compared to the control mice (25.7±5.2 s) in reaching the target position (**Figure 2G**). The frequency of the Cdk5f/f/KA1 mice crossing the platform was also significantly lower than controls (**Figure 2H**). No differences in swimming speeds were observed between Cdk5f/f/KA1 and Cdk5f/f mice (**Figure S2D**). These results suggest that Cdk5 is essential for pattern completion-based memory formation, a form of memory previously described as involving hippocampal CA3 circuits [29].

Collectively, the ablation of Cdk5 in hippocampal area CA1 impairs memory formation while the loss of Cdk5 function in hippocampal area CA3 affects pattern completion, consistent with previously established memory functions in these particular hippocampal circuits [25], [29]. No neuronal degeneration or dramatic morphological changes were observed in these mice (**Figure S1F, S2**). These results provide compelling evidence for an integral role of Cdk5 in regulating hippocampus-dependent memory functions. As synaptic plasticity and the signaling molecules involved in hippocampus-dependent memory events are best understood in area CA1, we utilized three-month-old Cdk5f/f/T29 mice for subsequent studies to dissect the downstream molecular pathway by which Cdk5 regulates learning and memory in the hippocampus.

### Impaired synaptic plasticity in Cdk5 mutant mice

**Figure 3:**
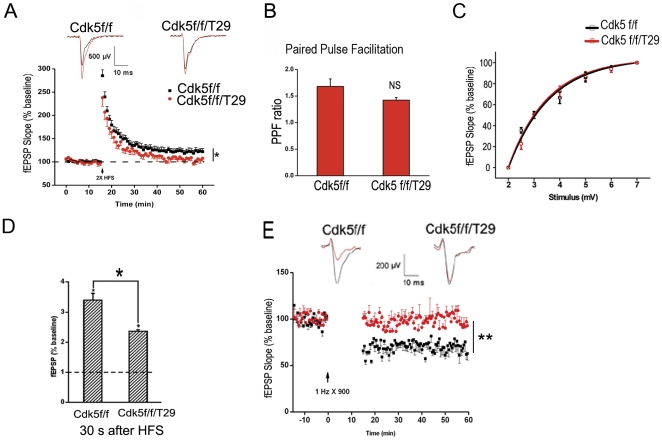
Synaptic plasticity is largely impaired in Cdk5f/f/T29 mice. **A**. Acute hippocampal slices obtained from 2-month-old Cdk5f/f/T29 and Cdk5f/f mice were stimulated with two trains of HFS (100 Hz, 1 s) in area CA1. Field EPSPs (fEPSPs) were measured from area CA1 in Cdk5f/f and Cdk5f/f/T29 mice (n = 8 for each group). **B**. Paired-pulse facilitation (PPF) in Cdk5f/f mice and Cdk5f/f/T29 and (n = 9 for each group). **C**. The input-output curve indicates normal basal synaptic transmission in the Cdk5f/f/T29 mice. **D.** Post-tetanic potentiation (PTP) from Cdk5f/f/T29 mice was lower than in Cdk5f/f mice 30 s after two trains of HFS (100 Hz, 1 s). **E**. LTD was measured in area CA1 of acute hippocampal slices from Cdk5f/f and Cdk5f/f/T29 after low frequency stimulation (1 Hz, 900 pulse; n = 6 for each group).

As synaptic plasticity has been implicated in memory formation, we therefore evaluated synaptic plasticity in the hippocampus of Cdk5f/f/T29 mice by examining long-term potentiation (LTP) in CA1 neurons. Acute transverse slices (400 µm) were obtained from Cdk5f/f/T29 or Cdk5f/f mice, and field excitatory postsynaptic potentials (fEPSPs) were evoked by placing an electrode on the Schaffer collateral pathway. After 15 min of baseline recording, LTP was induced using two trains of high-frequency stimulation (HFS; 100 Hz, 1 s). Forty-five minutes after HFS, field excitatory postsynaptic potentials (fEPSPs) remained elevated in the Cdk5f/f slices (121.88±7.04%; **Figure 3A**). In contrast, fEPSPs decayed to baseline in the Cdk5f/f/T29 slices (109.13±6.79%; **Figure 3A**, *p<0.01 Cdk5f/f *vs.* Cdk5f/f/T29). Presynaptic transmission was unimpaired, as measured by the paired-pulse facilitation (PPF) ratio, which measures the probability of presynaptic neurotransmitter release (**Figure 3B**). The input-output curves generated by plotting the fEPSP slopes against the slopes of the fiber volley were also indistinguishable, suggesting normal basal synaptic transmission (**Figure 3C**). However, Cdk5f/f/T29 slices exhibited defects in post-tetanic potentiation (PTP; **Figure 3D**). Although PTP is thought to be mainly affected by presynaptic mechanisms, postsynaptic injection of Ca^2+^ chelators, which affects postsynaptic cAMP activation, have also been found to block PTP [30]. Thus, neuronal Cdk5 ablation is associated with both LTP and PTP deficits in hippocampal CA1 neurons. We then proceeded to evaluate long-term depression (LTD). Low-frequency stimulation (LFS; 1 Hz, 900 pulses, 15 min) of the Schaffer collateral pathway induced a long-lasting synaptic depression in the Cdk5f/f animals. In contrast, LTD could not be induced in Cdk5f/f/T29 mice (**Figure 3E**). These data indicate that both LTP and LTD measures of synaptic plasticity are largely impaired in the Cdk5 mutant mice, which is consistent with their memory deficits.

### Disruption of CREB activity in Cdk5f/f/T29 mice

**Figure 4:**
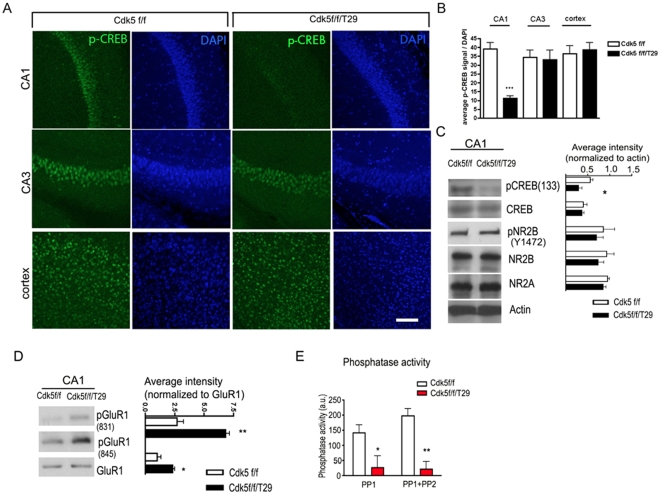
Impaired CREB phosphorylation and hyper-phorsphorylation of GluR1 in Cdk5f/f/T29 mice. **A–B**. Immunostaining (**A**) and quantification (**B**) of pCREB in hippocampal CA1 and cortex. Scale bar = 100 µm. **C**. Western blots of micro-dissected CA1 tissue show reduced pCREB levels. **D**. Western blots of micro-dissected CA1 tissue reveals hyper-phosphorylation of GluR1. **E.** Area CA1 phosphatase activity in Cdk5f/f and Cdk5f/f/T29 mice (n = 3, each group). *p<0.05, **p<0.005, ***p<0.001.

To dissect the molecular pathways involved in Cdk5-mediated synaptic plasticity and memory formation, we evaluated well-established signaling molecules involved in memory formation in the Cdk5f/f/T29 mice. CREB is an important transcription factor essential for expression of genes important for synaptic plasticity. A decrease in phosphorylated CREB on Serine 133 pCREB(S133), a modification required for CREB-mediated transcriptional activity during memory formation [31], was detected using immunohistochemistry on tissue sections prepared from mouse brain, as well as by immunoblots using an anti-pCREB(S133) antibody. The reduction of pCREB(S133) was specific to area CA1 neurons (**Figure 4A–B**), as no reduction of pCREB(S133) was detected in either cortical or CA3 neurons, where Cdk5 expression was unaffected. In lysates derived from hippocampal area CA1, pCREB(133) was markedly decreased in the Cdk5f/f/T29 compared to the Cdk5f/f mice (**Figure 4C**).

NMDA receptors are major mediators of synaptic plasticity; in a previously generated Cdk5 conditional knockout mouse model, it was demonstrated that NR2B degradation is impaired, resulting in increased surface expression of NR2B and enhanced synaptic plasticity [23]. The degradation of NR2B was reported to be regulated by phosphorylation of Tyrosine 1472 (Y1472) on NR2B via Cdk5 [32]. We found no differences in the protein levels of NR2A and NR2B in area CA1 between Cdk5f/f/T29 and Cdk5f/f mice (**Figure 4C**). Moreover, pNR2B (Y1472) was not altered in CA1 hippocampal lysates from Cdk5f/f/T29 mice compared to control littermates. Thus, the synaptic plasticity and learning behavior deficits observed in the Cdk5f/f/T29 mouse are associated with impaired CREB phosphorylation, but not with alterations in total NR2A or NR2B protein levels, or NR2B Y1472 phosphorylation.

The incorporation of GluR1-containing AMPA receptors into the synapse constitutes a major mechanism underlying the plasticity produced by the activation of CaMKII and LTP induction [33]. We found that the phosphorylation of GluR1 at S831, a site phosphorylated by CaMKII [34], was increased in Cdk5f/f/T29 samples compared to controls at basal levels (**Figure 4D**). In addition, phosphorylation of GluR1 at S845, a PKA site [34], was also upregulated (**Figure 4D**). Conversely, the activity of protein phosphatases 1 and 2 (PP1 and PP2) was markedly reduced in the Cdk5f/f/T29 mice (**Figure 4E**). Thus, decreased phosphatase activity may contribute to the increase in GluR1 phosphorylation. Taken together, these results indicate substantial perturbations in intracellular signaling pathways related to synaptic plasticity in the absence of Cdk5.

### Proteomic analysis reveals protein composition changes in Cdk5 mutant mice

**Figure 5:**
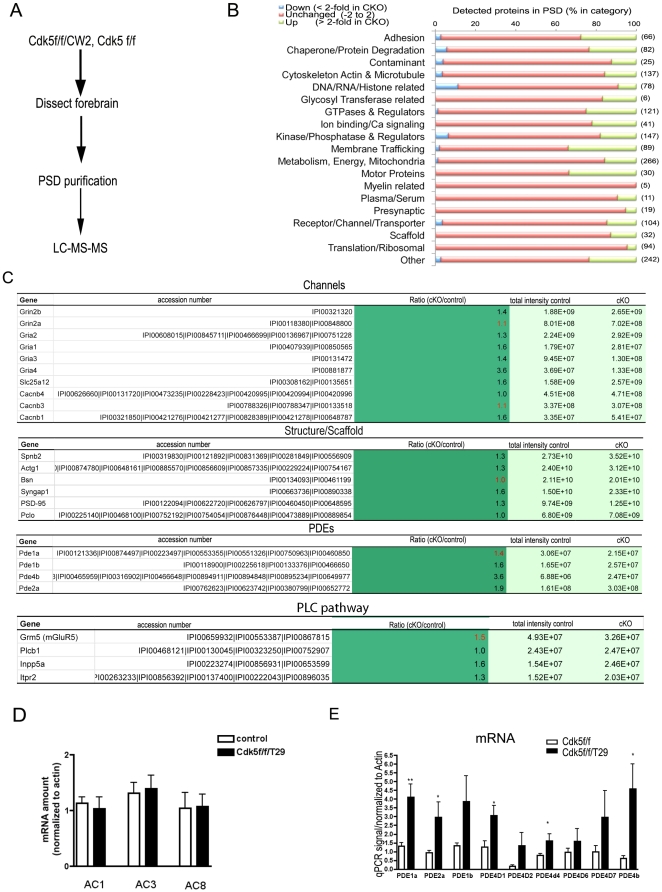
Proteomic analysis of post-synaptic densities reveals changes in PDE proteins in CDK5 mutant mice. **A**. Procedure sequence for mass-spectrometry analysis with PSDs from control (Cdk5f/f) and Cdk5f/f/CW2 (cKO) mice. **B**. Summary of detected protein abundance differences categorized by various protein functions. **C.** Representative molecules changes detected in Cdk5 cKO mice. Red number indicates reduction folds; black number indicates increase folds. **D**. RT-qPCR results of ACs in hippocampal CA1 in Cdk5f/f and Cdk5f/f/T29. qPCR signals were normalized to actin in each sample (n = 3). **E.** RT-qPCR results of PDEs from hippocampal area CA1 in Cdk5f/f and Cdk5f/f/T29.

To further decipher the mechanism by which Cdk5 influences synaptic plasticity, we applied a total, unbiased, proteomic approach to analyze changes in protein composition and phosphorylation in synaptosomal preparations of control and mutant mice. To this end, brains from control and Cdk5f/f/CW2 mice were homogenized and subjected to standard synaptosomal, postsynaptic density (PSD) isolation. Solubilized PSD proteins were run on an SDS-PAGE gel that was sliced into thirteen fields, and sequenced by LC/MS/MS (**Figure 5A** **, Figure S4A–B**). Approximately 1595 distinct, differentially expressed peptide groups were detected from control (Cdk5f/f) and Cdk5f/f/CW2 PSD preparations; of these, 700 peptides, implicated in dendritic spine/synapse formation, synaptic vesicle exocytosis, small GTPase signaling pathways, and ion channel functions, contain consensus Cdk5 phosphorylation sites. Among these potential Cdk5 substrates, the abundance of 296 proteins was increased, and the abundance of 51 proteins was decreased, in the Cdk5f/f/CW2 mice compared with controls (**Figure 5B**). These proteins comprised multiple different categories, including motor proteins and protein involved in membrane trafficking and kinase/phosphatase activity. Interestingly, consistent with the western immunoblotting results (**Figure 4C**), NMDA receptor subunits did not show significant changes (<1.5 fold) in the PSD fractions (**Figure 5C**; Grin2a, 2b). In contrast, multiple GluRs showed dramatic increase in the PSD fractions (**Figure 5C**; Gria1–4), suggesting increased synaptic AMPA receptors protein levels in the mutant mice. Overall, the drastic changes of protein composition in the PSD of Cdk5f/f/CW2 *vs*. controls suggest a key role of Cdk5 in regulating synaptic protein expression and abundance.

To further probe the mechanism underlying the phenotype of the Cdk5 conditional knock-out mice, we used our proteomic data to examine potential Cdk5 targets that could affect CREB activation. Among the known regulators of cAMP signaling pathway, cyclic nucleotide phosphodiesterase (PDE) proteins, enzymes that catalyze the conversion of cAMP and AMP into cGMP and GMP, respectively, were markedly increased in the Cdk5f/f/CW2 mice (**Figure 5C**). Conversely the adenylyl cyclase (AC) family proteins, which catalyze ATP to cAMP [35], were not detected in our proteomic data set, suggesting a low abundance of those proteins in the PSD preparation.

To further confirm the proteomic analysis, mRNA levels of AC family members were measured in control and mutant mice. Group 1 ACs are represented by AC1, AC3, and AC8, and are stimulated by calmodulin in a Ca^2+^-dependent manner [36]. We examined the mRNA levels of AC1, 3, and 8, but did not observe differences in the Cdk5f/f/T29 mice compared to controls (**Figure 5D**). However, we detected increased mRNA expression of PDE family proteins in the Cdk5 mutant mice using quantitative PCR with hippocampal RNA extracts. After signal normalization, mRNAs for PDE1a, PDE4D1, PDE4D4, PDE2a, and PDE4b were significantly increased in the Cdk5f/f/T29 mouse hippocampus compared to Cdk5f/f (**Figure 5E**). PDE4 proteins exclusively target cAMP and is a specific target for rolipram, while PDE1 and 2 also target cGMP. While cAMP has been shown to regulate LTP [11], [37], cGMP is involved in regulating LTD [38], The increase in the PDE1 and 2 isoforms, which deplete cGMP, in Cdk5f/f/T29 mice might be a contributing factor to the loss of LTD. Thus, increased PDE expression in the Cdk5f/f/T29 mice may impair LTP and LTD induction via depletion of cAMP and cGMP.

### Cdk5 loss of function blocks activation of the cAMP activation pathway, which can be restored by rolipram

**Figure 6:**
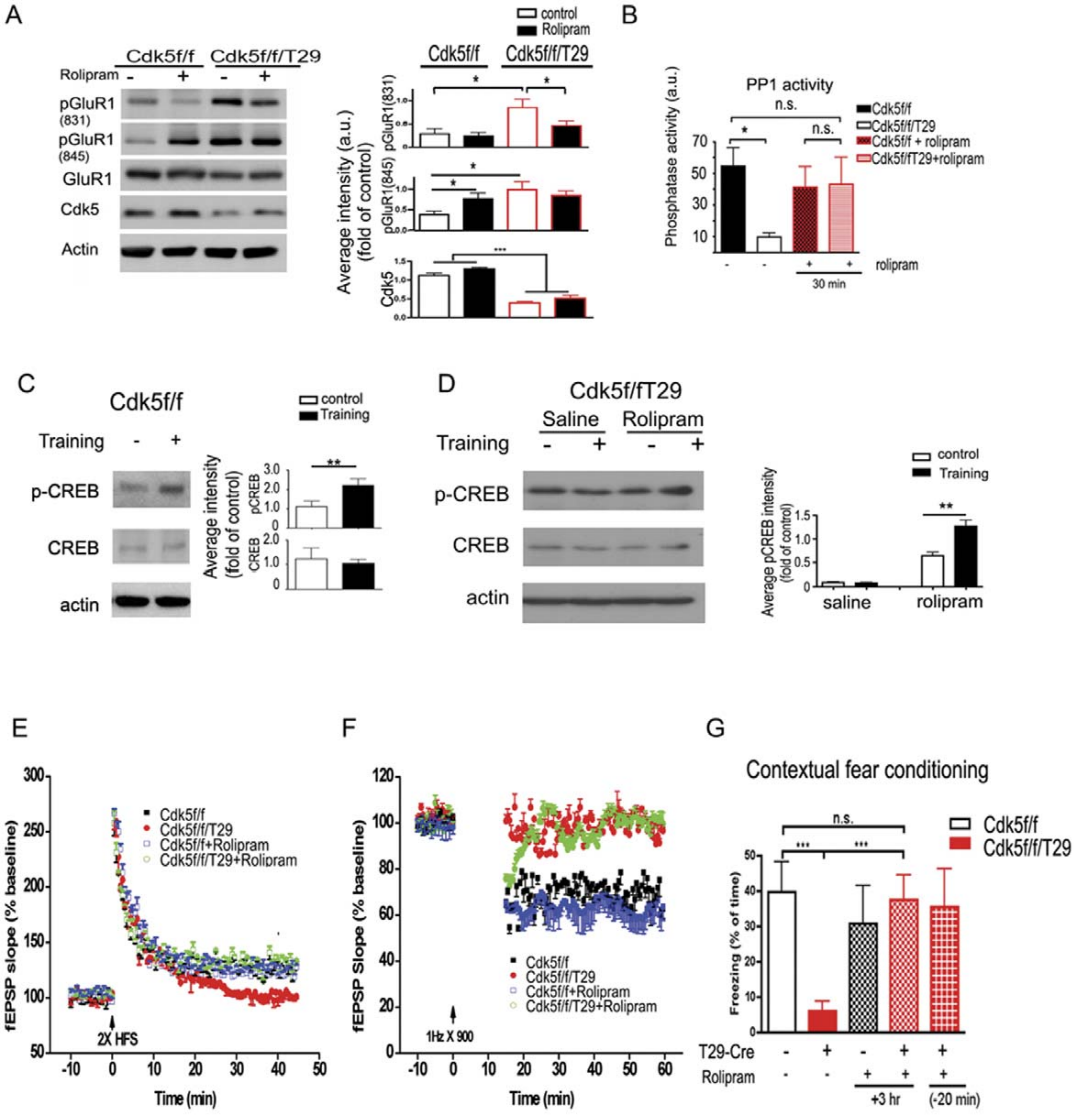
Inhibition of PDE4 by rolipram can restore LTP deficits and rescue memory impairments in Cdk5f/f/T29 mice. **A**. Western blot from CA1 lysates after rolipram treatment. Samples were collected 20 min after rolipram or saline (control) injection. **B**. PP1 activity in area CA1 tissue was measured after rolipram treatment (0.1 mg/kg, i.p.). **C**. Western blots (left) for CA1 tissues from Cdk5f/f mice, with quantification (right). Mice were trained in a contextual fear conditioning paradigm followed by tissue harvest 20 min after training. n = 5 for each group. **D**. Western blots (left) from micro-dissected CA1 tissues from Cdk5f/f/T29 mice with quantification (right). Rolipram (0.1 mg/kg, i.p.) was injected 20 min before contextual fear conditioning training. Samples were collected 20 min after training (n = 3 for each group). **E**. Rolipram restored LTP deficits in the Cdk5f/f/T29 slices. Mice were injected with rolipram or vehicle (Veh) 20 min before sacrifice and slice preparation. Rolipram was also present in the bath solution at 0.7 µM. Slices were stimulated with two trains of HFS (100 Hz, 1 s; n = 6 for each group; *p<0.05, between vehicle- and rolipram-treated groups in Cdk5f/f/T29 mice). **F**. Rolipram did not rescue the LTD impairment in Cdk5f/f/T29 slices (n = 5 for each group). **G**. In a contextual fear conditioning task, the percent of time spent freezing in Cdk5f/f/T29 mice (n = 16) is significantly lower than that of control Cdk5f/f mice (n = 8). The percent freezing time for the Cdk5f/f/T29 mice injected with rolipram (0.1 mg/kg, i.p.) 20 min before training (n = 8) or 3 hr after training (n = 7) is similar to that of the control Cdk5 f/f mice.

To further test the hypothesis that the dysregulated cAMP signaling, by virtue of increased PDE expression, underlies the observed plasticity and behavioral phenotypes exhibited by the Cdk5 mutant mice, we evaluated the consequences of the pharmacological inhibition of these enzymes in the Cdk5f/f/T29 mice. Rolipram, a PDE4-specific small molecule inhibitor, was recently reported to facilitate memory formation and synaptic plasticity in rodents [13]. In Cdk5f/f mice, rolipram treatment resulted in the increased phosphorylation of GluR1 at S845, consistent with a role for cAMP signaling and PKA phosphorylation of S845 (**Figure 6A**). At basal levels, phosphorylation of GluR1 at both S831 and 845 in Cdk5f/f/T29 mice is significantly higher compared to Cdk5f/f controls. However, rolipram treatment in Cdk5f/f/T29 mice selectively reduces phosphorylation of GluR1 at S831, indicating that the inhibition of PDE4 may affect GluR1 phosphorylation preferentially at the CaMKII phosphorylation site. To further examine if PDE inhibition, and therefore persistent cAMP signaling, has influences on other biochemical changes in Cdk5f/f/T29 mice, we evaluated PP1 activity. As PDEs are upregulated in the Cdk5 mutant mice, we explored the consequences of PDE inhibition by rolipram. Interestingly, blocking PDE4 activity by rolipram (0.1 mg/kg, i.p.) in Cdk5f/f/T29 mice restores PP1 activity (**Figure 6B**).

We next evaluated the effect of rolipram pretreatment on the activity-induced phosphorylation of CREB(S133) in the Cdk5f/f/T29 mouse. Rolipram (0.1 mg/kg) was administered into Cdk5f/f or Cdk5f/f/T29 mice 20 min before contextual fear conditioning training. Animals were sacrificed 20 min after training and hippocampal area CA1 was dissected for biochemical processing. Training increased pCREB(S133) in area CA1 of Cdk5f/f mice (**Figure 6C**), whereas the saline-injected Cdk5f/f/T29 group did not show any increase in pCREB(S133) levels after training (**Figure 6D**). However, in rolipram-treated Cdk5f/f/T29 mice, contextual fear training induced a significant increase in pCREB in hippocampal area CA1, suggesting that rolipram treatment rescued the training-induced activation of pCREB (**Figure 6D**). Thus, Cdk5 enabled the activation of pCREB via the regulation of cAMP levels.

### Rolipram treatment partially restores synaptic plasticity in Cdk5f/f/T29 mice

To determine if rolipram treatment influences synaptic plasticity, Cdk5f/f and Cdk5f/f/T29 mice were injected with rolipram (0.1 mg/kg) 20 min before the preparation of acute hippocampal slices for LTP and LTD recordings. Rolipram (0.7 µM) was present in the bath solution throughout the entire experiment. Cdk5f/f slices treated with rolipram alone did not demonstrate enhanced LTP induction compared to Cdk5f/f treated with saline (**Figure 6E** **)**. Consistent with our previous observations (**Figure 3A**), Cdk5f/f/T29 mice treated with saline exhibited deficits in LTP. Remarkably, following HFS stimulation, fEPSPs remained potentiated (123.2±5.7%) in the hippocampal slices prepared from the rolipram-treated Cdk5f/f/T29 slices, comparable to the level of LTP induced in Cdk5f/f slices. Thus, inhibition of PDE4 by rolipram rescues LTP in the Cdk5f/f/T29 mice. In contrast, although fEPSPs were initially depressed in the rolipram-treated Cdk5f/f/T29 mice after LFS stimulation for LTD induction, synaptic depression was transient and rapidly decayed to baseline levels (**Figure 6F**). Thus, rolipram treatment is sufficient to restore LTP, but not LTD, in the Cdk5f/f/T29 mice. This is also consistent with a reduced phosphorylation of GluR1 on the CaMKII (S831) site, but not the PKA (S845) site [6], in the untrained mice.

### Rolipram treatment rescues associative memory in Cdk5f/f/T29 mice

We next investigated whether rolipram could extend its ameliorative effects to the memory impairment caused by Cdk5 loss of function in hippocampal area CA1. Using the contextual fear conditioning paradigm, we found that rolipram administration (0.1 mg/kg) either 20 min before, or 3 hr after, training rescued the memory impairments in the Cdk5f/f/T29 mice (**Figure 6G**). When tested 24 hr after training, the average freezing time of Cdk5f/f/T29 mice was 5.9±2.6%. However, the average freezing time of Cdk5f/f/T29 mice receiving rolipram increased to 35.4±10.8% (the group treated 20 min before training) and 37.3±7.1% (the group treated 3 hr after training) respectively, which were comparable to the average freezing time of control mice (39.6±8.6%; **Figure 6G**). Rolipram treatment in control animals did not alter freezing behavior (30.6±11.0% *vs.* 39.6±8.6%) in the contextual fear conditioning tasks (**Figure 6G**). The rescue of contextual fear memory consolidation with rolipram treatment is especially remarkable considering the severity of the Cdk5f/f/T29 behavioral phenotype, in which untreated mice fail to express contextual fear memory even after three consecutive days of training (**Figure 1F**).

## Discussion

### Cdk5 is essential for hippocampal-dependent learning and memory

Cdk5 is thought to be involved in the morphogenesis of synaptic connections, as well as in the regulation of synaptic transmission, through interactions at both presynaptic terminals and postsynaptic spines with proteins that include PSD-95 [39], ErbB [40], Stat3 [41], DARPP-32 [42], CASK [43], and Eph4/ephexin1 [44]. In contrast to an earlier study by Hawasli et al in which enhanced hippocampal synaptic plasticity and spatial learning behaviors were observed due to upregulated NMDA receptor subunit NR2B levels [23], we find that the neuron-specific Cdk5 loss of function has a detrimental effect on hippocampal-dependent associative and spatial memory, and impairs synaptic plasticity, but leads to unaltered NR2B levels. The differences between the previously reported findings and our current observations can be attributed to the methods used to generate the different Cdk5 knockout mouse models. In the previous Cdk5 conditional knockout study [23], the use of the prion promoter extends Cdk5 knockdown to general regions of the brain including the forebrain, striatum, cerebellum, and hippocampus [45], [46], [47]. The prion promoter also drives expression in various neural cell types, including glial cells in which Cdk5 is also expressed [46]–[50]. Thus, the regional loss of Cdk5 in different cell types may account for previous behavioral observations. Another distinction between these studies consists of the acute *vs.* the chronic deletion of Cdk5, given the findings that chronic loss of Cdk5 in the inducible mouse model (8 weeks after ablation of Cdk5) resulted in significantly different behavior compared to the acute loss of Cdk5 function (2–4 weeks) [51]. Therefore, while data from the prion promoter mouse model suggest that the widespread acute loss of Cdk5 in both neurons and glia is beneficial for cognition, chronic ablation of Cdk5 in hippocampal circuits led to learning impairments in our Cdk5 models prior to the spread of the Cre promoter expression. This suggests that the targeted deletion of Cdk5 in excitatory neurons of hippocampal areas CA1 and CA3 or forebrain is associated with impaired cAMP signaling, synaptic plasticity, and memory formation. We noticed a slight reduction of Cdk5 in the dentate gyrus of Cdk5f/f/KA1 mice (**Figure 2C**). Therefore, it is possible that Cdk5 may play a role in the DG that affects the observed behavioral phenotypes. In future experiments, it would be of interest to examine how the loss of Cdk5 in other brain regions (such as the DG), or in specific cell-types, including dopaminergic neurons, cholinergic neurons, or a subset of interneurons, impacts the various neural circuits and signaling pathways by which Cdk5 regulates memory formation.

The same strategy used to generate Cdk5 conditional knockout mice in the current work was previously used in studies targeting the NR1 gene to study synaptic plasticity and evaluate the role of the NMDA receptor in areas CA1 and CA3 of the hippocampus [25], [29]. The similarities between our findings and those using CA1- and CA3-targeted NR1 knockout mice support the notion that Cdk5 is involved in the CA1 spatial memory formation circuit, and in the CA3 pattern completion-based memory recall circuit. However, the memory deficit in the Cdk5f/f/T29 mice appears to be more severe than in mice lacking the NR1 receptor subunit in the CA1 region, as reflected by their poor performance on contextual fear conditioning tasks, even following repeated training. During the Morris water maze hidden platform training, the Cdk5f/f/T29 mice did not exhibit any decrease in average escape latencies despite repeated trainings. Our findings indicate that the more severe phenotype evident in Cdk5f/f/T29 may result from the involvement of Cdk5 in the regulation of cAMP pathways and synaptic transmission via the regulation of phosphatases and PDEs. Thus, Cdk5-mediated regulation of the cAMP pathway is required and essential for memory formation.

### Cdk5 modulates PDE signaling pathways essential for synaptic plasticity

Mechanistically, the memory deficits caused by the ablation of Cdk5 are not due to developmental influences, as the postnatal nature of Cre expression with the T29-Cre line has been well-characterized [24], [52], and hippocampal morphology is not severely altered in any of our Cdk5 mutant mice. The learning phenotype also cannot be attributed to neurodegeneration, as there are no significant differences in the number of pyramidal neurons between the Cdk5-deficient mice and controls (**Figure S1F**). Instead, we identified an abnormal increase in the expression of several PDEs in the brain, suggesting that the cAMP pathway is impacted in the Cdk5 mutant mice. Furthermore, PDE inhibition by acute rolipram treatment restored LTP and CREB phosphorylation in Cdk5f/f/T29 mice. Importantly, rolipram treatment ameliorated the memory deficits observed in the Cdk5f/f/T29 mice. These data strongly support a novel role for Cdk5 in regulating memory via the cAMP pathway.

Earlier mouse genetic studies have elegantly demonstrated that increases in CREB phosphorylation, and subsequent CREB-mediated gene transcription, occur following contextual fear conditioning. Conversely, disrupting CREB leads to deficiencies in transcription-dependent long-term memory in mice [53], [54]. The impaired cAMP signaling observed in Cdk5-deficient mice in our study is accompanied by a reduction in pCREB(S133) in the hippocampus in both basal and post-training conditions. Interestingly, the Cdk5f/f/T29 mutant mice demonstrated an early LTP impairment, a phenomenon independent of CREB-mediated transcription modifications [55]. It should be noted that CREB is not the only target and effecter of cAMP, and earlier studies have shown that postsynaptic cAMP pathways directly regulate early LTP in the hippocampal CA1 area [37]. Our results support the essential role of the cAMP pathway in modulating synaptic functions as well as in learning and memory.

We propose that the alteration of the cAMP pathway, resulting from increased PDE expression, is a major factor in the aberrant signaling pathways and behavior deficits observed in Cdk5-deficient mice (**Figure S5**). However, the detailed mechanism by which Cdk5 regulates PDEs remains unclear. Cdk5 might act upon multiple targets to regulate the levels of intracellular cAMP. We found that both the mRNA and protein levels of PDE4 are increased in Cdk5 mutant mice, which could result from a lack of inhibition by Cdk5 on targeted transcription factors such as MEF2 [56]. In the Cdk5 mutant mice, we also detected a lack of pDARRP-32(T75) (data not shown), which would inhibit PKA signaling [42], [57] and could indirectly affect cAMP levels via feedback regulation through the AC-cAMP-PKA axis [58]. Other possibilities include a direct regulation on the activity of AC by Cdk5, which cannot be excluded as an alternative interpretation based on our experimental evidence.

### Other potential synaptic mechanisms regulated by Cdk5

Interestingly, in our PSD proteomic screen, many PSD molecules in pathways other than the cAMP pathway are altered in the absence of Cdk5, including receptor, channel, and trafficking molecules. For example, GluR4 and the L-type calcium channel, alpha 2, myo5a, Kif2a are increased, while ACCN1, GABA receptor type 2, and dynll2 are decreased, in the PSD of Cdk5 mutant mice (**Figure 5C**). The alterations in PSD protein composition resulting from the loss of Cdk5 suggest that Cdk5 is an active participant at the synapse whose critical role in synaptic plasticity may be mediated via more than one distinct pathway.

In the current work, we also identified altered AMPAR-dependent regulation, as measured by changes in GluR1 phosphorylation. In Cdk5f/f/T29 mice, we observed increased GluR1 phosphorylation at both S831 and S845 (**Figure 4D**). Partially increasing GluR1 phosphorylation at S831 might facilitate LTP [5], [6], while the occlusion of GluR1 phosphorylation by the long-term deletion of Cdk5 is predicted to lead to a failure in LTP induction. The mouse models used in this study may therefore represent a chronic loss of Cdk5 in adult mice in which LTP occlusion is derived from persistent GluR1 phosphorylation. Intriguingly, rolipram treatment in the Cdk5f/f/T29 mice decreased the S831, but not S845, GluR1 phosphorylation and restored LTP, but not LTD, signaling. As PP1 phosphatase activity is also restored to control levels with rolipram application, one could speculate that PP1 regulates the bidirectional GluR1 signaling and LTP by the dephosphorylation of S831. However, as LTD is not restored in Cdk5f/f/T29 mice treated with rolipram, Cdk5 may act through a PDE4-independent pathway, such as the PDE1 and PDE2 pathways that deplete cGMP, in regulating LTD maintenance. Alternatively, Cdk5 may play a role in generating LTD that is separate from PDE function altogether.

### Directions for future research

In the current work, we have elucidated a novel role for Cdk5 in the modulation of the cAMP pathway. We cannot exclude the possibility that other targets of Cdk5 may also be involved in the regulation of cAMP, such as the direct phosphorylation of PDE4 or AC by Cdk5. Future experiments include addressing the relationships between Cdk5 activity and intracellular signaling molecules such as cAMP or PP1 as key regulators of activity-dependent mechanisms of memory formation, along with work examining how GluR1 phosphorylation is regulated by Cdk5 and how this contributes to pathways by which Cdk5 maintains downstream signaling at the glutamatergic synapse. Recent evidence has highlighted Cdk5 as a critical molecule implicated in the downregulation of synaptic activity in hippocampal neurons [59] and in the regulation of synaptic transmission [60]. Further work on the molecular targets of Cdk5, including cAMP, will reveal the mechanisms by which Cdk5 maintains synaptic homeostasis.

The modulation of the PDEs and cAMP pathways has already been utilized to treat mouse models of cognitive decline, as rolipram was previously shown to be able to rescue the memory deficits in mouse models of Rubinstein-Taybi Syndrome [15], ischemia [61], and Alzheimer's disease [13], [62]. Our findings that rolipram ameliorates the memory deficits caused by ablation of Cdk5 in hippocampal CA1 neurons provides a compelling direction for future research in targeting PDEs and other cAMP pathway activators to restore memory deficits, particularly those associated with neurodegeneration and cognitive decline.

## Materials and Methods

### Animals

Animals were derived from the C57BL/6J strain obtained from Jackson Labs (Bar Harbor, ME) due to their common use in genetic crossing and maintaining genetically modified mouse lines. The mice were group housed in the small animal facility at the Department of Brain and Cognitive Sciences of Massachusetts Institute of Technology (Cambridge, MA) with a 12-hr light dark cycle and ad libitum feeding.

### Ethics Statement

All experiments were conducted in compliance with the humane animal care standards outlined in the NIH Guide for the Care and Use of Experimental Animals and were reviewed and approved by the MIT Committee on Animal Care (CAC). The protocol number is 0609-060-12, approval date July 27, 2010.

### Generation of region-specific Cdk5 mutant mice

We constructed a targeting vector in which two loxP sequences were inserted into the Cdk5 gene. The first loxP sequence was placed before exon 1 and the second loxP sequence was placed between exons 5 and 6 so that they flanked exons 1–5 of the twelve total exons in the Cdk5 genomic sequence. The mice homozygous for the loxP-Cdk5-loxP sequence (henceforth named “floxed Cdk5” or simply ‘Cdk5f/f’) were generated through standard homologous recombination procedures (**Figure S1A–B**). The Cdk5-loxP construct was linearized by Pme1 digestion and electroporated into embryonic stem cells. The homologous recombinant clones were identified by Southern blot (see next section). The chimeric mice were crossed to Flp transgenic mice (Susan Dymecki) to remove the neomycin cassette. Floxed mice were genotyped using the PCR primer sequences: (forward) cagtttctagcacccaactgatgta and (reverse) gctgtcctggaactccatctataga cagtttctagcacccaactgatgta and reverse: gctgtcctggaactccatctataga. Floxed Cdk5 mice were maintained on a C57BL/6 background.

For region-specific ablation of Cdk5, we used mouse lines predominantly expressing Cre in excitatory neurons of hippocampal areas CA1 or CA3 and forebrain. We crossed Cdk5f/f mice with Cre transgenic mice, T29-2, which mediate Cre/loxP recombination predominantly in CA1 pyramidal cells [24], [25] to generate Cdk5f/f/T29 mice (Cre^T29-2^;fCdk5/fCdk5). Cdk5f/f/CW2 mice were generated by crossing the CW-2 Cre mice to the Cdk5f/f mice [28] in order to ablate Cdk5 in excitatory neurons of the forebrain. To create Cdk5f/f/KA1 mice, we crossed Cdk5f/f mice with Cre transgenic mice, G32-4, which express Cre predominantly in CA3 pyramidal cells [3]. We confirmed CA1, forebrain, and CA3 Cre expression pattern by crossing the Cre lines to the ROSA26-EYFP line [63]. For the following experiments, we used Cdk5 homozygous floxed littermates (Cdk5f/f) as controls. Cdk5f/f/T29, Cdk5f/f/CW2, and Cdk5f/f/KA1 mice are viable and do not exhibit any obvious developmental defects, in contrast to the embryonic lethality of Cdk5 KO mice produced by conventional gene knockout strategies [64]. We confirmed the ablation of Cdk5 in areas CA1 or CA3 by immunohistochemistry. Cdk5 is expressed in most areas of the mouse brain. In the hippocampus, we found that Cdk5 expression level in area CA1 was decreased in Cdk5f/f/T29 mice compared to controls. Importantly, there were no differences in Cdk5 expression levels between the Cdk5f/f/T29 and control mice in area CA3 and dentate gyrus (DG). Cdk5 immunoreactivity in CA3 was reduced in the Cdk5f/f/KA1 mice compared to controls. Cdk5 was absent in the cell bodies of pyramidal cells in area CA1 of the Cdk5f/f/T29 mice and in area CA3 of Cdk5f/f/KA1 mice. Morphological and histochemical examinations did not reveal obvious abnormalities in brains from Cdk5f/f/T29, Cdk5f/f/CW2, or Cdk5f/f/KA1 mice (**Figures S1, S2, S3**). Tissues collected from micro-dissected area CA1 of the Cdk5f/f/T29 versus control mice (Cdk5f/f) revealed that Cdk5 protein levels are largely reduced in the CA1 region of these mice.

### Southern blotting

The following modified protocol was used: tail lysis overnight, followed by DNA extraction. Ten µg of genomic DNA was digested with BamHI and EcoRI overnight, followed by electrophoresis on a 0.8% agarose gel. The gel was then incubated in 2 gel volumes of 0.25 M HCl for 20 min at room temperature, briefly rinsed with ddH2O, then incubated in 2 gel volumes of denaturation buffer (0.5 M NaOH, 1.5 M NaCl) two times, 15 min each. The gel was briefly rinsed with ddH_2_O and subsequently incubated in 2 gel volumes of renaturation buffer two times, 30 min each. The transfer was set up overnight on a sandwich consisting of 3 cm high paper towel stack, the agarose gel, a nitrocellulose membrane (Micron Separation, Inc.), and filter paper with a bridge. The membrane was then rinsed and baked in a 80**°**C oven under a vacuum for 2 hr, rinsed 2 times in 2X SSC buffer (for 1 L 20X SSC: 3 M NaCl, 0.3 M Sodium Citrate, pH 7.0), and prehybridized in ULTRAHTb (Ambion) for 1 hr at 42°C in the hybridization chamber. The probe was made using a DECAprime II kit (Ambion, Cat #1455). The reaction was incubated at 37**°**C for 7 min, terminated by 1 µl 0.5M EDTA, and unincorporated nucleotides were removed using spin columns (NuAway, Ambion). Radiolabel incorporation was measured using a scintillation counter. 107 cpm per ml of probe was transferred to the prehybridized blot and incubated overnight at 42**°**C. The membrane was then washed sequentially: 2X SSC, 0.1% SDS for 20 min at 65°C, 0.1X SSC, 0.1% SDS for 20 min three times at 65**°**C. The blot was then dried and developed with film.

### Behavior

Adult (2.5–3.5 month old) mice were used for all behavior, biochemical, and immunohistochemistry studies. We observed that the Cre expression in the T29-2 line is no longer restricted to the CA1 region in 4-month-old mice. Interestingly, the spreading of Cre expression in these mice, and thus Cdk5 knockdown, occurs at a time when the Cdk5f/f/T29 mice begin to suffer seizures, and these mice begin to die around 6–8 months of age. Cdk5f/f/KA1 mice appear to be normal even after 1 year of age.

### Contextual fear conditioning

Training consisted of a 3 min exposure of mice to the conditioning box (context) followed by a foot shock (2 sec, 0.5/0.8/1.0 mA, constant current). The memory test was performed 24 hr later by re-exposing the mice to the conditioning context for 3 min. Freezing, defined as a lack of movement except for heartbeat and respiration associated with a crouching posture, was recorded every 10 sec by two trained observers (one was blind to the experimental conditions) during the 3 min trial for a total of 18 sampling intervals. The mean number of observations indicating freezing from both observers was expressed as a percentage of the total number of observations.

### Cued fear conditioning

Training consisted of a 3 min exposure of mice to the conditioning box, followed by a tone (30 sec, 20 kHz, 75 dB sound pressure level (SPL) and a foot shock (2 sec, 0.8 mA, constant current) [65]. The memory test was performed 24 hr later by exposing the mice for 3 min to a novel context followed by an additional 3 min exposure to a tone (20 kHz, 75 dB SPL). Freezing was recorded every 10 sec by two trained observers as described above.

### Morris water maze

The water maze with hidden platform paradigm [1] was performed in a circular tank (diameter 1.8 m) filled with opaque water. A platform (11×11 cm) was submerged below the water's surface in the center of the target quadrant. The swimming path of the mice was recorded by a video camera and analyzed by the Videomot 2 software (TSE). For each training trial, the mice were placed into the maze consecutively from one of four random points of the tank. Mice were allowed to search for the platform for 60 s. If the mice did not find the platform within 60 s, they were gently guided to it. Mice were allowed to remain on the platform for 15 s. Two consecutive training trials were given every day; the latency for each trial was recorded for analysis. During the memory test (probe test), the platform was removed from the tank, and the mice were allowed to swim in the maze for 60 s.

### Immunohistochemistry and immunoblotting

Immunohistochemistry was performed as described previously [66]. Briefly, brains were fixed in 4% paraformaldehyde and stained with antibodies against Cdk5 and pCREB. Five random fields from each experiment were obtained and quantified with the observer blind to genotype. The antibodies and dilutions used were as follows. For immunohistochemistry: Cdk5 (MBS240590, MyBioSource, 1∶800); pCREB(S133) (Millipore 06-519, 1∶200). Immunoblotting was performed on mouse forebrains or microdissected hippocampi. Brain lysates were obtained by dounce homogenization of tissue in radioimmunoprecipitation (RIPA) buffer. The RIPA buffer composition is as follows: 50 mM Tris pH 8, 150 mM NaCl, 1% NP−40, 0.5% sodium deoxycholate, 0.1% SDS with protease and phosphatase inhibitor tablets (Roche). Lysates were incubated on ice and cleared with a 13,000 rpm spin and protein content was quantified (BCA protein assay, Bio-Rad Technologies). Ten micrograms of protein was diluted with 2X sample buffer consisting of the following: 100 mM Tris pH 6.8, 4% SDS (w/v), 0.2% bromophenol blue (w/v), 20% glycerol (v/v), 200 mM DTT. Samples were boiled at 95**°**C for 5 minutes and resolved on a 10% SDS-polyacrylamide gel with 8% stacking gels using Laemmli buffer. Proteins were transferred by electrophoresis using tris-glycine wet transfer onto PVDF membranes (Millipore) for 1 hr on ice. After blocking with blocking buffer (5% non-fat dry milk/0.1% Tween-20/TBS) for 1 hr, membranes were probed with various antibodies overnight at 4**°**C. Membranes were washed three times using 0.1% Tween-20/TBS, incubated with secondary antibodies (enhanced chemiluminescence mouse or rabbit IgG, HRP-Linked F(ab')2 fragment from sheep, GE Healthcare, diluted at 1∶15,000) for 1 hr at room temperature. Membranes were washed again and developed using Western Lightning ECL substrate (Perkin Elmer). All antibodies were diluted in blocking buffer. Membrane stripping (before reprobing) was performed with stripping buffer: (2%SDS, 62.5 mM Tris-HCL pH 6.8, 100 mM 2-mecaptoethanol) and incubation for 30 minutes at 50**°**C (with rocking), followed by two washing steps with excess TBST, and blocking as usual. Immunoblots were quantified using ImageJ (NIH). Statistical analysis was performed using Prism software. Antibodies used for immunoblots: Cdk5 (DC-17, Tsai laboratory, 1∶500); pCaMKII(T286) (Cell Signaling Technology #3361, 1∶2000); pCREB(S133) (Millipore 06-519, 1∶2000); pGluR1(S845) (Chemicon Ab5849); pGluR1(S831) (Chemicon, Ab5847); GluR1 (Abcam); pPP1 (Cell Signal 2581); pp1a (Abcam); DARRP-32 T75 (Cell Signal #2301); DARRP-32 T34 (Cell Signal #2304); actin (Sigma #5316).

### Phosphatase assay

Whole hippocampi were dissected and homogenized in 3.75 mM Tris-HCl, pH 7.4, 15 mM KCl, 3.75 mM NaCl, 250 µM EDTA, 50 µM EGTA, 30% (w/v) sucrose, 30% (v/v) glycerol, protease inhibitor cocktail (Sigma), 100 µM PMSF using a Dounce homogenizer, then centrifuged (1000 g, 10 min). For each sample, the supernatant (cytoplasmic fraction) and pellet (nuclear fraction) were separated. Each fraction was resuspended in the same buffer without sucrose, but including 15 mM PMSF, using a Dounce homogenizer then triturated with a 26 G syringe before purification on PiResin (Innova Biosciences). Phosphatase activity was determined by incubating 2 µL sample with 0.15 mM RII substrate (BIOMOL) and 5 nM tautomycin (to inhibit PP1) or 5 nM tautomycin + OA (to inhibit PP1 and PP2A activity) in 50 mM Tris-HCl, pH 7.0, 100 µM Na2EDTA, 5 mM DTT, 0.01% Brij35 at 30°C for 10 min. The reaction was terminated by adding TCA followed by centrifugation (13,000 g, 5 min). The amount of free phosphates released in the reaction was measured with BIOMOL Green reagent (BIOMOL) at 620 nm and background-subtracted. For total phosphatase activity, tautomycin and OA were removed from the reaction. PP1 and PP2A activity was calculated using the ratio of phosphatase activity with inhibitors and total phosphatase activity.

### Electrophysiology

Three to four month old Cdk5f/f/T29 mice or their Cdk5f/f control littermates were sacrificed by cervical dislocation, and hippocampi were rapidly dissected in ice-cold oxygenated artificial CSF (ACSF). Transverse hippocampal slices (400 µm thick) were placed in a chamber and continuously perfused with oxygenated ACSF consisting of (in mM): 119 NaCl, 2.5 KCl, 2.5 CaCl_2_, 1.3 MgSO_4_, 1 NaH_2_PO_4_, 26.2 NaHCO_3_, 11 D-glucose, pH 7.4. A bipolar stimulating electrode (0.002 in diameter nichrome wire; A-M Systems) placed in the stratum radiatum was used to elicit action potentials in CA3 Schaffer collateral axons. An ACSF-filled glass microelectrode with a resistance between 0.5 and 3 MΩ was placed in the stratum radiatum region of area CA1 and was used to record the field excitatory post-synaptic potentials (fEPSP). Data were acquired using HEKA EPC10 and analyzed by Patchmaster (HEKA). Peak fEPSP amplitudes from stimulators were required to be at least 2 mV, and stimulus intensity was set to produce 40% of the maximal response. Baseline responses were recorded for 20 min. fEPSPs were evoked at area CA1 synapses by stimulating Schaffer collaterals at a low frequency (2 per min) to establish a stable baseline. Immediately following LTP induction with 2 trains of high-frequency stimulation (HFS, 100 Hz, 1 s), with an interval of 20 s, slices from Cdk5f/f/T29 and control Cdk5f/f mice showed an increase in fEPSP slope and amplitude, suggesting that short-term potentiation (STP) occurs in all groups.

### PSD preparation

Forebrains from Cdk5f/f and Cdk5f/f/CW2 mice were homogenized and postsynaptic densities (PSD) isolated in ice-cold buffers with protease and phosphatase inhibitor cocktails (Roche) as previously described with minor modifications [67]. Briefly, mouse forebrains were isolated and homogenized in ice-cold Buffer A (0.32 M Sucrose, 6 mM Tris (pH 8), 1 mM MgCl_2_, 0.5 mM CaCl_2_) with a Teflon homogenizer (15 strokes). Homogenized brain extracts were spun at 1400×g for 10 min. The supernatant (S1) was saved and the subsequent pellet (P1) was homogenized again (5 strokes). After centrifugation at 700×g, the supernatant (S1') was saved and pooled with S1. Pooled S1 and S1' was centrifuged at 13,800×g for 10 min to collect the pellet (P2). P2 was resuspended in Buffer B (0.32 M Sucrose, 6 mM Tris pH 8; 5 strokes). The P2 suspension was loaded onto a discontinuous sucrose gradient (0.85 M/1 M/1.15 M sucrose solution in 6 mM Tris, pH 8.0), followed by centrifugation for 2 h at 82,500×g in a SW-41 rotor. The synaptosome fraction between 1 M and 1.15 M sucrose was collected with a syringe needle and adjusted to 4 ml with Buffer B. Equal volumes of Buffer C (12 mM Tris pH 8; 1% Triton X-100) was added and mixed for 15 min and then spun at 32,800×g in a Ti70.1 rotor for 20 min. The PSD-enriched pellet was resuspended in 40 mM Tris (pH 8) and protein concentration was measured using a BCA assay. To prepare samples for mass spectrometry, solubilized PSD proteins were reduced with 10 mM DTT for 30 min at 60°C. After cooling to room temperature, the sample was then alkylated with 25 mM iodoacetamide for 30 min at room temperature. After adding 2X sample buffer (100 mM Tris pH 6.8, 200 mM DTT, 4% SDS, 0.2% Bromophenol blue, 20% glycerol), the PSD samples were then boiled at 95°C for 5 min and 30 µg of each sample was loaded for separation on an 4–12% SDS-PAGE gradient gel (Invitrogen).

### In-gel Digestion and Mass Spectrometry

Lanes from the gel were excised, cut into 13 fields as shown in Figure S4B and digested overnight at 37°C with an excess of sequencing grade trypsin. Peptides were extracted from the gel with 50% acetonitrile/0.1% trifluoroacetic acid and concentrated in a Speed-Vac. Tryptic digests were analyzed with an automated nano LC-MS/MS system, consisting of an Agilent 1100 nano-LC system (Agilent Technologies, Wilmington, DE) coupled to an LTQ-Orbitrap Fourier transform mass spectrometer (Thermo Fisher Scientific, San Jose, CA) equipped with a nanoflow ionization source (James A. Hill Instrument Services, Arlington, MA). Peptides were eluted from a 10 cm column (Picofrit 75 µm ID, New Objectives) packed in-house with ReproSil-Pur C18-AQ 3 µm reversed phase resin (Dr. Maisch, Ammerbuch Germany) using a 90 min acetonitrile/0.1% formic acid gradient at a flow rate of 200 nl/min to yield ∼15 s peak widths. The elution portion of LC gradient was 3–7% solvent B in 2 min, 7–37% in 58 min, 37–90% in 3 min, and held at 90% solvent B for 5 min. Data-dependent LC-MS/MS spectra were acquired in ∼3 s cycles; each cycle was of the following form: one full Orbitrap MS scan at 60,000 resolution followed by 8 MS/MS scans in the ion trap on the most abundant precursor ions using an isolation width of 3 m/z. Dynamic exclusion was enabled with a mass width of ±25 ppm, a repeat count of 1, and an exclusion duration of 12 sec. Charge state screening was enabled along with monoisotopic precursor selection to prevent triggering of MS/MS on precursor ions with unassigned charge or a charge state of 1. Normalized collision energy was set to 30 with an activation Q of 0.25 and activation time of 30 ms.

### Protein identification, quantification, and phosphosite determination

All MS data was interpreted using the using the Spectrum Mill software package v4.0 beta (Agilent Technologies, Santa Clara, CA). Similar MS/MS spectra acquired on the same precursor m/z within ±60 sec were merged, and poor quality MS/MS spectra which failed the quality filter of having a sequence tag length >0 (i.e., minimum of two masses separated by the in-chain mass of an amino acid) were excluded from searching. MS/MS spectra were searched against the International Protein Index (IPI) mouse database version 3.48. Initial search parameters included: ESI linear ion trap scoring parameters, trypsin enzyme specificity with a maximum of two missed cleavages, 30% minimum matched peak intensity, ±20 ppm precursor mass tolerance, ±0.7 Da product mass tolerance, and carbamidomethylation of cysteines as a fixed modification. Allowed variable modifications were oxidized methionines, deamidation of asparagine, and pyro-glutamic acid modification at N-terminal glutamines with a precursor MH+ shift range of −18 to 65 Da. Identities interpreted for individual spectra were automatically designated as valid by applying the scoring threshold criteria provided below to all spectra in a two-step process. First, protein mode was used, which requires two or more matched peptides per protein while allowing a range of medium to excellent scores for each peptide. Second, peptide mode was applied to the remaining spectra, allowing for excellent scoring peptides that are detected as the sole evidence for particular proteins. Protein mode thresholds: protein score >20, peptide (score, Scored Percent Intensity, delta rank1 - rank2) peptide charge +2: (>8, >65%, >2) peptide charge +3: (>9, >65%, >2) peptide charge +4: (>9, >70%, >2) peptide charge +2: (>6, >90%, >1). Peptide mode thresholds: peptide charge +2 and +3 (>13, >70, >2) peptide charge +4 (>15, >70, >2) respectively. The above criteria yielded a false discovery rate of <1% as estimated by target-decoy based searches using reversed sequences. MS/MS spectra of phosphopeptides were interpreted in a second round of searches against only the subset of proteins confidently identified from the unphosphorylated peptides observed during the initial round of searches. The allowed variable modifications were expanded to include phosphorylated serine, threonine, and tyrosine, with a precursor MH+ shift range of −18 to 177 Da (no more than 2 phosphosites/peptide). The spectrum of each phosphopeptide was manually inspected by an expert. For ∼10% of phosphosites observed the MS/MS spectra lack sufficient information to assign the site of phosphorylation to a particular Ser, Thr, or Tyr residue.

The relative abundances of proteins were determined using extracted ion chromatograms (XICs) for each peptide precursor ion in the intervening high resolution FT-MS scans of the LC-MS/MS runs. An individual protein's abundance was calculated as the sum of the ion current measured for all quantifiable peptide precursor ions with MS/MS spectra confidently assigned to that protein. Peptides were considered not quantifiable if they were shared across multiple subgroups of a protein or the precursor ions had a poorly defined isotope cluster (i.e. the “subgroup specific” and “exclude poor isotope quality precursor” XIC filters in Spectrum Mill were enabled). Proteins were considered not quantifiable if there were fewer than two distinct peptides observed in either the control or cKO samples. Since equivalent amounts of total protein were loaded in each lane of the gel, and both samples were subsequently treated equivalently, no further normalization was done when calculating protein abundance ratios between the two samples. The peak area for the XIC of each precursor ion subjected to MS/MS was calculated automatically by the Spectrum Mill software in the intervening high-resolution MS1 scans of the LC-MS/MS runs using narrow windows around each individual member of the isotope cluster. Peak widths in both the time and *m*/*z* domains were dynamically determined based on MS scan resolution, precursor charge, and *m*/*z* subject to quality metrics on the relative distribution of the peaks in the isotope cluster *vs.* theoretical. Although the determined protein ratios are generally reliable to within a factor of two-fold of the actual ratio, numerous experimental factors contribute to variability in the determined abundance for a protein. These factors may include incomplete digestion of the protein; widely varying response of individual peptides due to inherent variability in ionization efficiency as well as interference/suppression by other components eluting at the same time as the peptide of interest, differences in instrument sensitivity over the mass range analyzed, and inadequate sampling of the chromatographic peak between MS/MS scans.

### Quantitative RT-PCR primers

mus AC1: cagcaggaaccaaggctaag; tggccacattgactgtgttt

mus AC3: tgaggagagcatcaacaacg; tggtgtgactcctgaagctg

mus AC8: ggactgtccccagagaaaca; cttactcccgtgctgtccat

mus pde1A: catgattgggttccatgttg; cagccaactctttccacctc

mus pde1b: tgcccttctctccactctgt; tgggctgacttttaggcttg

mus PDE2A: gaccgatggagatgatggac; acttgtgggacaccttggtc

mus PDE4D1: tatgaaggagcagccctcatg; ccaggacatcttcctgctctg

mus PDE4D4: tggccagtttctggtaggcctc; gagctacccgtggtcgctac

mus PDE4D6: ccaggacatcttcctgctctg; cacattttagaacttgctgtcac

mus PDE4D7: tggccagtttctggtaggcctc; actactcaaaaccgcaccatgg

mus PDE4B: ggaaaaatcccaggttggtt; cagtccctgctcctctcatc

## Supporting Information

Figure S1 **Generation of mouse models with Cdk5 ablation in hippocampal areas CA1 and CA3. A**. Cre/loxP recombination system design Cdk5 conditional knockouts. Exons 1–5 of CDK5 are flanked with loxP sites. **B**. Southern blot indicating the correct insertion of loxP sites. **C**. The Cre/loxP strategy designed for cell-type-restricted Cdk5 knockout in area CA1 (Cdk5f/f/T29) or forebrain (Cdk5f/f/CW2). The Cre expression is driven by the αCaMKII promoter which is found in excitatory neurons **D**. Immunostaining for GFP in the hippocampus of reporter CRE^T29-2^/R26Sor mice. The T29-2 Cre line (area CA1-specific CRE line) was crossed to the reporter line R26Sor. Scale bar = 1 mm. **E**. Immunostaining for GFP in the hippocampus of reporter CRE^CW2^/R26Sor. The CW2 Cre line (forebrain-specific CRE line) was crossed to the reporter line R26Sor. **F.** Representative pictures showing NeuN labeling in the hippocampus of Cdk5f/f, Cdk5f/f/T29 and Cdk5f/f/KA1 mice (3 month old). No obvious neuronal loss was seen in the different groups. **G.** Open field test for Cdk5f/f and Cdk5f/f/T29 mice. No differences were observed during the first 5 min of activity in the open field. **H**. Swimming speeds for Cdk5f/f and Cdk5f/f/T29 mice during the Morris water maze task. No significant difference was observed**. I.** Western immunoblot showing the reduction of Cdk5 in the forebrain of Cdk5f/f/CW2 mice and quantification (n = 3 mice per group).
(TIF)

Figure S2 **Examination of neuronal morphology and cell death in Cdk5 mutant mice. A.** Cdk5f/f (control) and Cdk5f/f/CW2 mice were stained with DAPI and imaged in area CA1 of the hippocampus, the cortex, and thalamus. No overall differences in neuronal morphology or cell death were observed. Scale bars, 100 µm. **B.** Immunoblots from Cdk5f/f and Cdk5f/f/T29 micro-dissections of area CA1 of the hippocampus do not reveal any activated cell death markers as assayed by cleaved caspase-3.
(TIF)

Figure S3 **Region-specific recombination in the hippocampus. A**. Immunostaining for GFP in the hippocampus of reporter CRE^G32-4^/R26Sor. G32-4 Cre mice (CA3-specific CRE line, denoted as Cdk5f/f/KA1) were crossed to the reporter line R26Sor. Scale bar = 1 mm. **B.** the generation of Cdk5f/f/KA1 mice in which Cdk5 is depleted in hippocampal area CA3. **C.** Immunostaining for Cdk5 in areas CA3 and DG of control mice Cdk5f/f and Cdk5f/f/KA1. Scale bar = 100 µm. **D**. Swimming speeds for Cdk5f/f and Cdk5f/f/KA1 mice. No significant differences were observed.
(TIF)

Figure S4 **Mass-spectrometry preparation of PSD samples. A.** Western immunoblotting of PSD proteins after preparation. Cdk5 is significantly reduced in forebrain PSD preparations of Cdk5f/f/CW2 mice. **B.** Coomassie blue staining of PSD proteins on an SDS-PAGE gel. Thirteen gel slices were obtained from each lane in preparation for LC/MS/MS.
(TIF)

Figure S5 **Cdk5 regulates homeostasis of the cAMP pathway.** Model figure demonstrating the proposed role of Cdk5/p35 in regulating the cAMP pathway and synaptic plasticity. Under normal conditions, activity stimulates cAMP and PKA activity, leading to altered cAMP-dependent changes in gene transcription and memory formation. Loss of Cdk5 severely attenuates cAMP signaling and impairs memory formation due to increased PDE activity. Rolipram, a PDE4 inhibitor, prevents the breakdown of cAMP and restores the signaling pathway mediating synaptic plasticity and memory formation.
(TIF)
