# Supplementary figures and images for: Cdk5 Is Required for Memory Function and Hippocampal Plasticity via the cAMP Signaling Pathway
Source: PLoS One. 2011 Sep 30;6(9):e25735. doi: 10.1371/journal.pone.0025735 (PMC3184170; doi:10.1371/journal.pone.0025735)

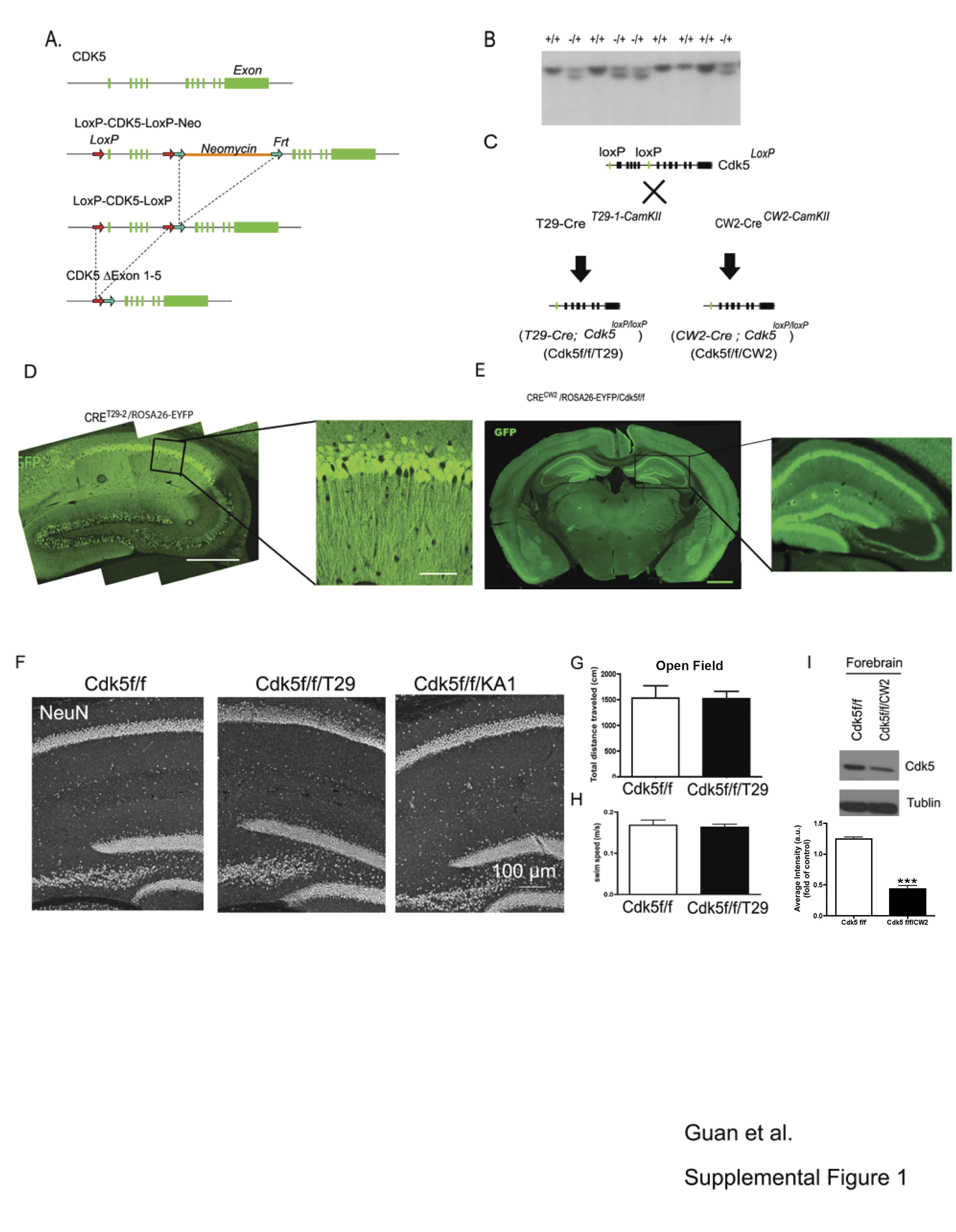

Supplement: Figure S1 — Generation of mouse models with Cdk5 ablation in hippocampal areas CA1 and CA3. A. Cre/loxP recombination system design Cdk5 conditional knockouts. Exons 1–5 of CDK5 are flanked with loxP sites. B. Southern blot indicating the correct insertion of loxP sites. C. The Cre/loxP strategy designed for cell-type-restricted Cdk5 knockout in area CA1 (Cdk5f/f/T29) or forebrain (Cdk5f/f/CW2). The Cre expression is driven by the αCaMKII promoter which is found in excitatory neurons D. Immunostaining for GFP in the hippocampus of reporter CRET29-2/R26Sor mice. The T29-2 Cre line (area CA1-specific CRE line) was crossed to the reporter line R26Sor. Scale bar = 1 mm. E. Immunostaining for GFP in the hippocampus of reporter CRECW2/R26Sor. The CW2 Cre line (forebrain-specific CRE line) was crossed to the reporter line R26Sor. F. Representative pictures showing NeuN labeling in the hippocampus of Cdk5f/f, Cdk5f/f/T29 and Cdk5f/f/KA1 mice (3 month old). No obvious neuronal loss was seen in the different groups. G. Open field test for Cdk5f/f and Cdk5f/f/T29 mice. No differences were observed during the first 5 min of activity in the open field. H. Swimming speeds for Cdk5f/f and Cdk5f/f/T29 mice during the Morris water maze task. No significant difference was observed. I. Western immunoblot showing the reduction of Cdk5 in the forebrain of Cdk5f/f/CW2 mice and quantification (n = 3 mice per group). (TIF) [file pone.0025735.s001.tif]

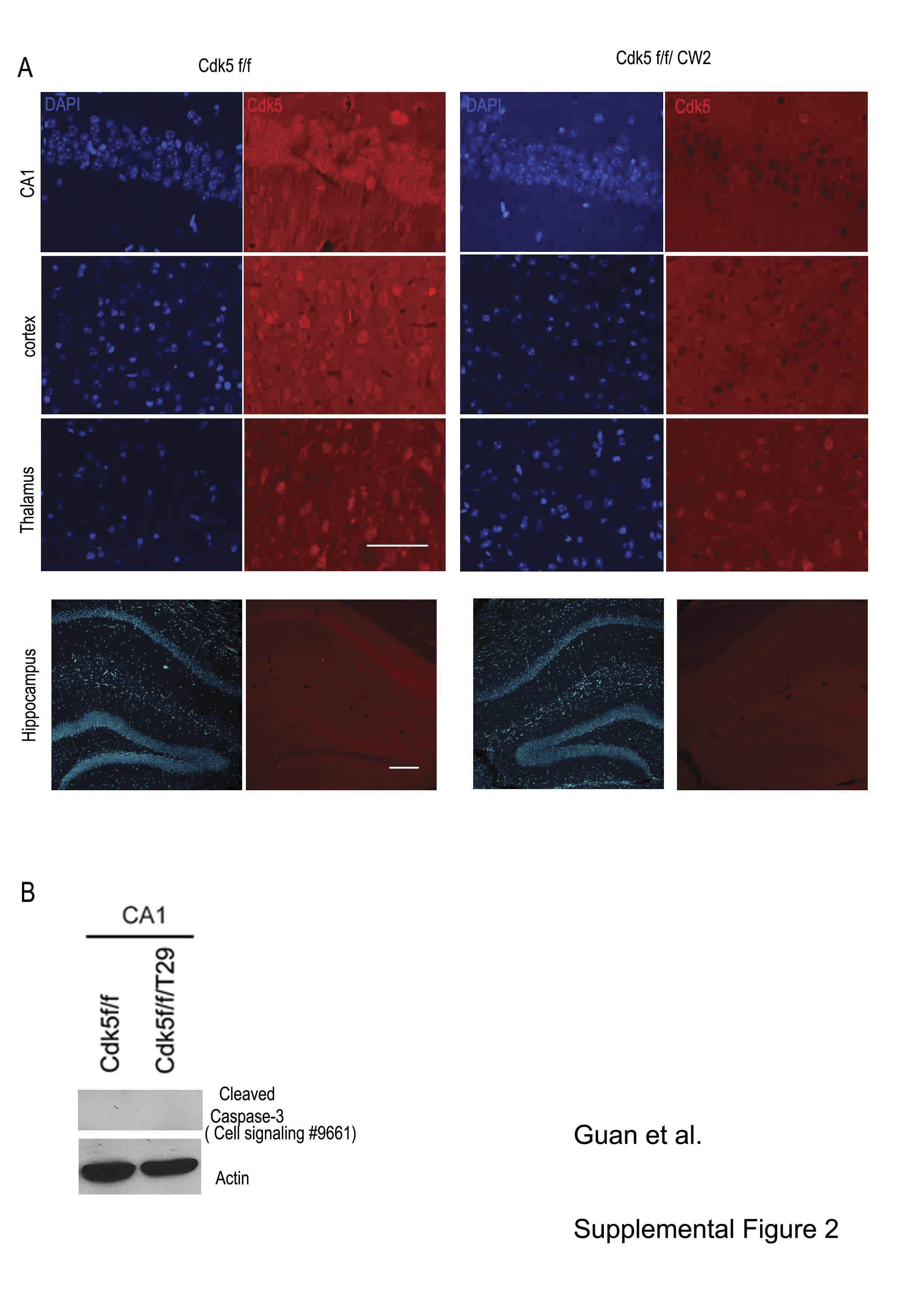

Supplement: Figure S2 — Examination of neuronal morphology and cell death in Cdk5 mutant mice. A. Cdk5f/f (control) and Cdk5f/f/CW2 mice were stained with DAPI and imaged in area CA1 of the hippocampus, the cortex, and thalamus. No overall differences in neuronal morphology or cell death were observed. Scale bars, 100 µm. B. Immunoblots from Cdk5f/f and Cdk5f/f/T29 micro-dissections of area CA1 of the hippocampus do not reveal any activated cell death markers as assayed by cleaved caspase-3. (TIF) [file pone.0025735.s002.tif]

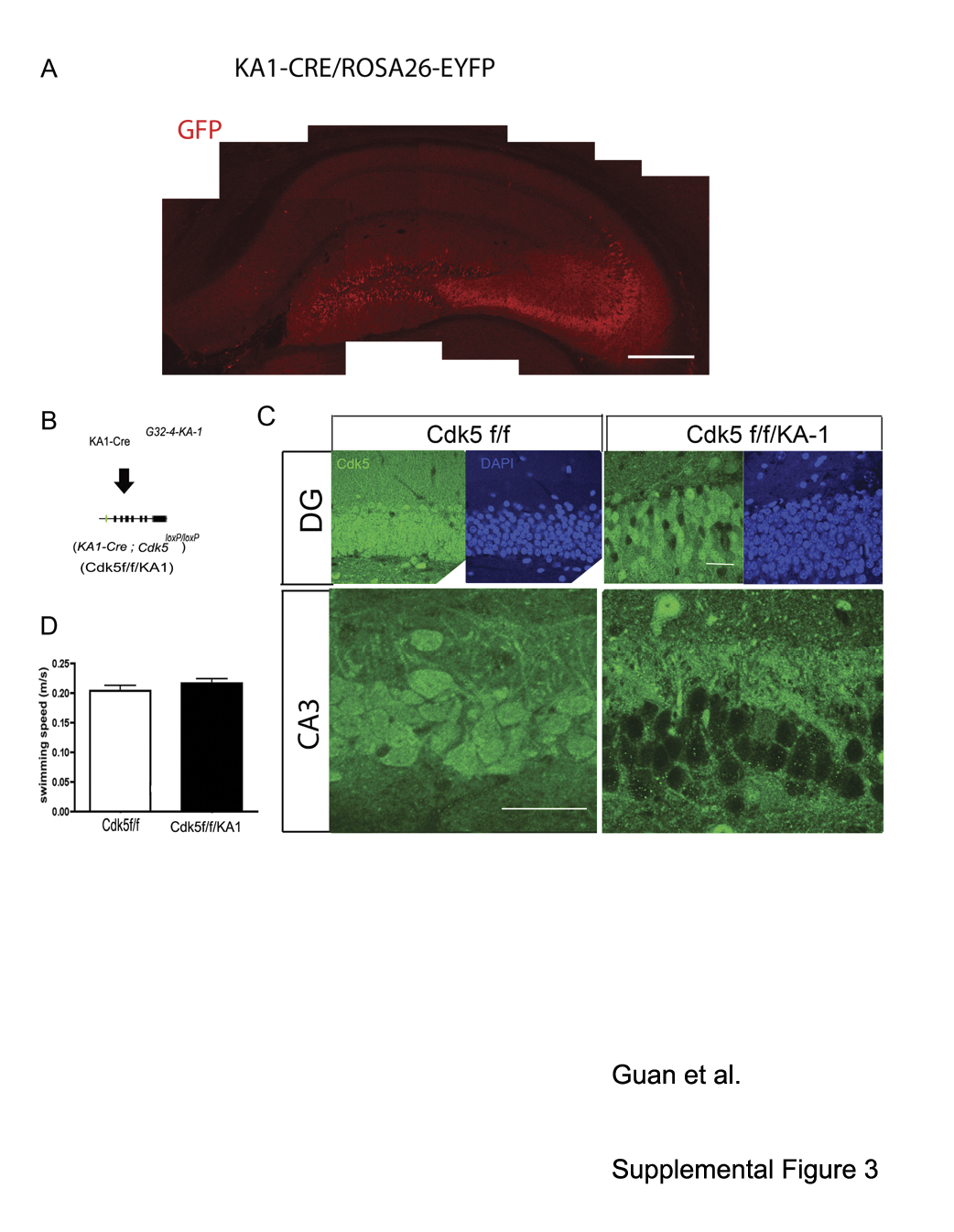

Supplement: Figure S3 — Region-specific recombination in the hippocampus. A. Immunostaining for GFP in the hippocampus of reporter CREG32-4/R26Sor. G32-4 Cre mice (CA3-specific CRE line, denoted as Cdk5f/f/KA1) were crossed to the reporter line R26Sor. Scale bar = 1 mm. B. the generation of Cdk5f/f/KA1 mice in which Cdk5 is depleted in hippocampal area CA3. C. Immunostaining for Cdk5 in areas CA3 and DG of control mice Cdk5f/f and Cdk5f/f/KA1. Scale bar = 100 µm. D. Swimming speeds for Cdk5f/f and Cdk5f/f/KA1 mice. No significant differences were observed. (TIF) [file pone.0025735.s003.tif]

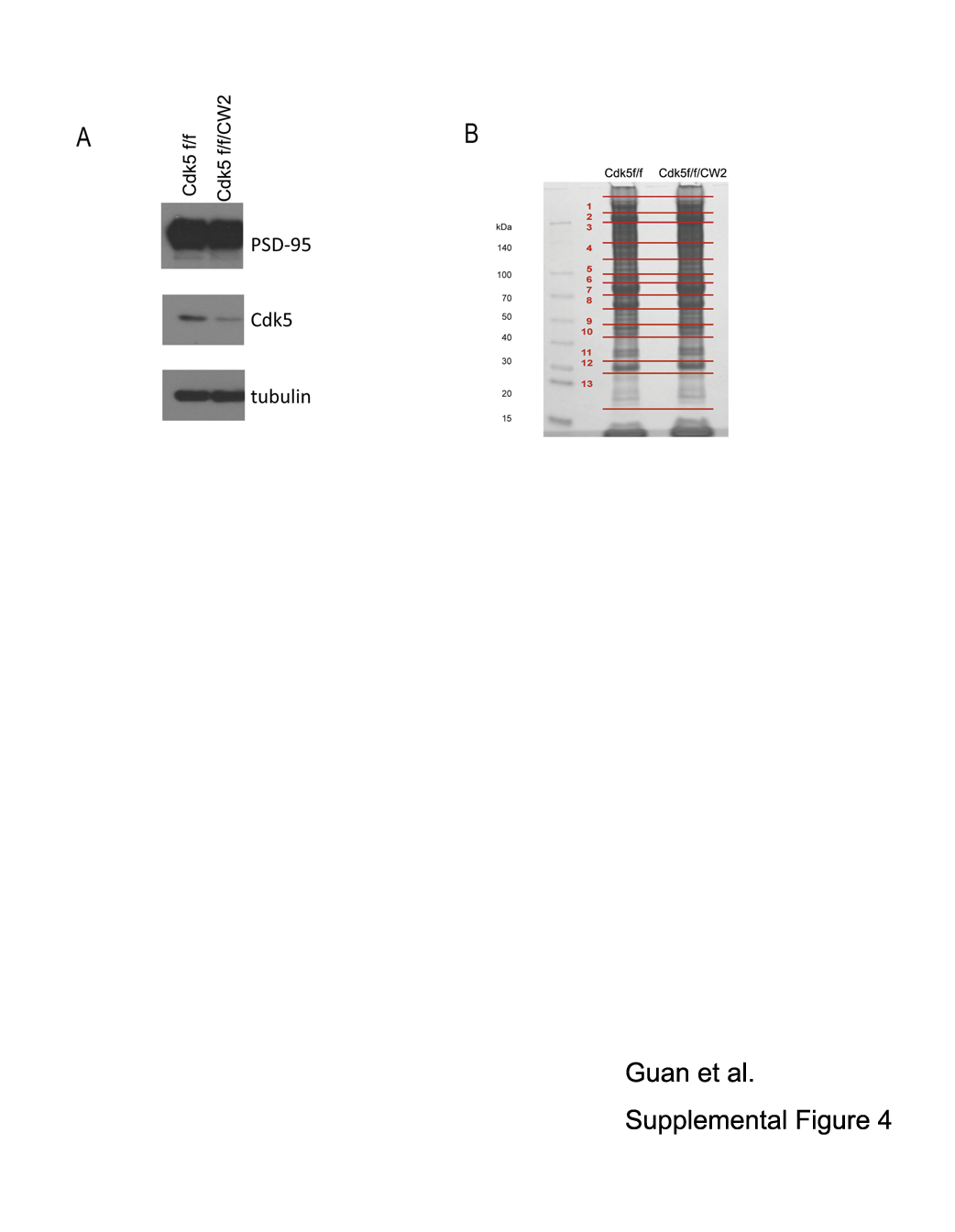

Supplement: Figure S4 — Mass-spectrometry preparation of PSD samples. A. Western immunoblotting of PSD proteins after preparation. Cdk5 is significantly reduced in forebrain PSD preparations of Cdk5f/f/CW2 mice. B. Coomassie blue staining of PSD proteins on an SDS-PAGE gel. Thirteen gel slices were obtained from each lane in preparation for LC/MS/MS. (TIF) [file pone.0025735.s004.tif]

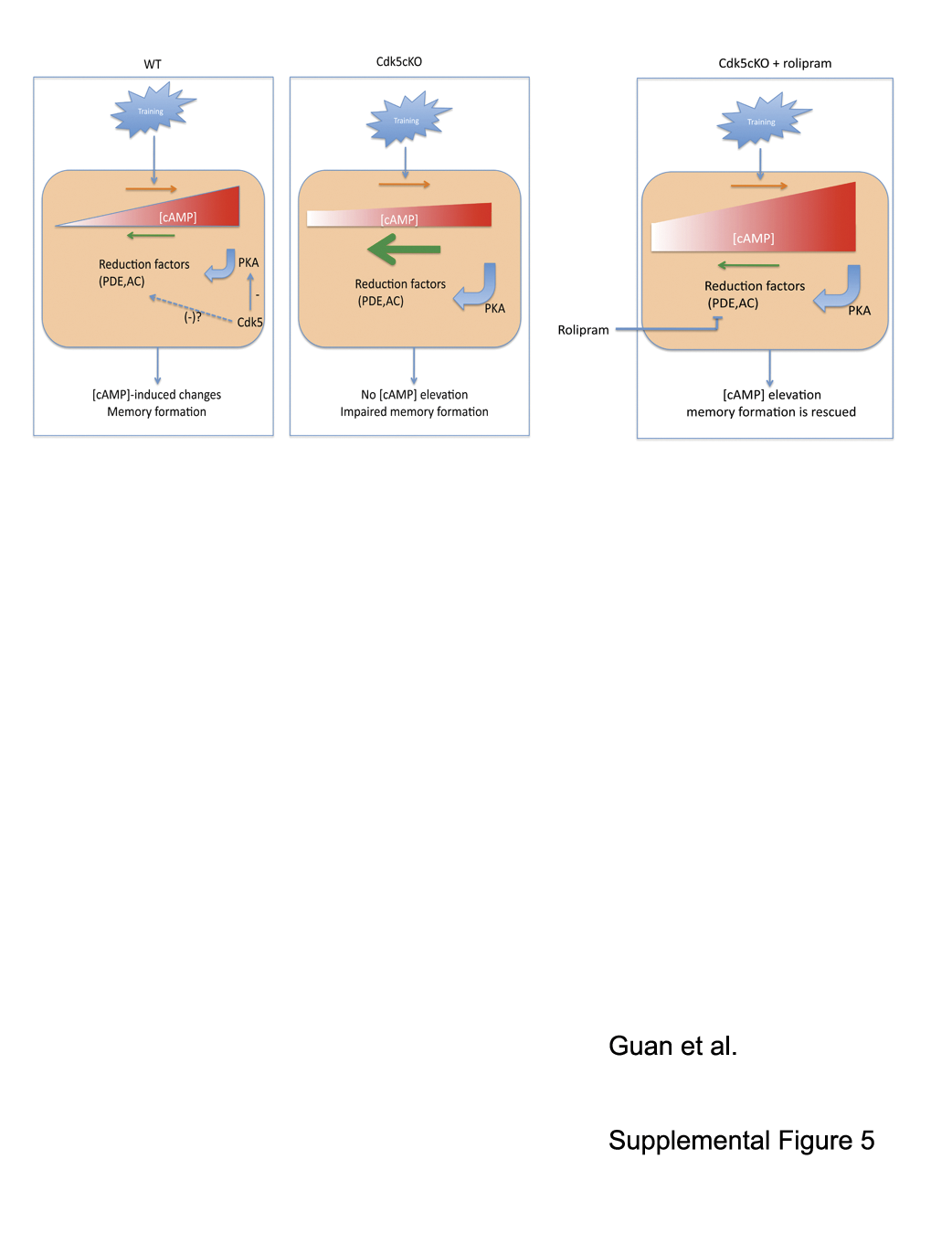

Supplement: Figure S5 — Cdk5 regulates homeostasis of the cAMP pathway. Model figure demonstrating the proposed role of Cdk5/p35 in regulating the cAMP pathway and synaptic plasticity. Under normal conditions, activity stimulates cAMP and PKA activity, leading to altered cAMP-dependent changes in gene transcription and memory formation. Loss of Cdk5 severely attenuates cAMP signaling and impairs memory formation due to increased PDE activity. Rolipram, a PDE4 inhibitor, prevents the breakdown of cAMP and restores the signaling pathway mediating synaptic plasticity and memory formation. (TIF) [file pone.0025735.s005.tif]
